# Numerical Analysis and Comparison of Three Iterative Methods Based on Finite Element for the 2D/3D Stationary Micropolar Fluid Equations

**DOI:** 10.3390/e24050628

**Published:** 2022-04-29

**Authors:** Xin Xing, Demin Liu

**Affiliations:** College of Mathematics and System Sciences, Xinjiang University, Urumqi 830046, China; xingxin@stu.xju.edu.cn

**Keywords:** micropolar fluid equations, finite element method, iterative methods, stability, error estimation

## Abstract

In this paper, three iterative methods (Stokes, Newton and Oseen iterative methods) based on finite element discretization for the stationary micropolar fluid equations are proposed, analyzed and compared. The stability and error estimation for the Stokes and Newton iterative methods are obtained under the strong uniqueness conditions. In addition, the stability and error estimation for the Oseen iterative method are derived under the uniqueness condition of the weak solution. Finally, numerical examples test the applicability and the effectiveness of the three iterative methods.

## 1. Introduction

The micropolar fluid equations can be used to describe the flow behavior of polymeric fluids. Based on the classical Navier–Stokes equations, Eringen [1] first proposed the micropolar fluid equations in 1965. The micropolar fluid equations postulate that every particle not only has mass and velocity but also has microinertia and microrotation, and can support not only stress, but couple stress. Micropolar fluids have been widely used in modern industry, biology, engineering and other fields. The dynamic behavior of fluid flow, such as particle suspensions, liquid crystals, lubrication and animal blood can be described by micropolar fluid equations [2,3].

Many scholars are devoted to study the well-posedness of solutions for the micropolar fluid equations. Galdi and Rionero [4] discussed the existence and uniqueness of weak solutions for the initial boundary value problem of the micropolar fluid equations. Rojas-medar and Boldrini [5] proved the global existence of weak solutions by the Galerkin method. Łukaszewicz [6,7] proved the local existence of strong solutions and the global existence of weak solutions by using the linearization method and fixed point theorems. Dong and Chen [8] proposed the regularity criteria of weak solutions for the 3D micropolar fluid equations.

In the past decades, the finite element method (FEM) has been widely used to solve the fluid dynamics equations. He and Li [9] introduced the three iterative methods for Navier–Stokes equations, and derived stability conditions for different iterative algorithms. Dong et al. [10] discussed and analyzed the three classical iterative methods for MHD equations. Based on the asymptotic behavior of the perturbation solutions of the steady Navier–Stokes equations, He [11] proposed the Euler implicit/explicit iterative scheme and proved the corresponding stability condition. In order to improve the calculation efficiency of Navier–Stokes equations, Xu [12], Xu and He [13] considered the two-level methods based on the iterative solutions. Layton [14,15] proposed and analyzed the two-level method to solve the nonlinearity. Huang et al. [16] proposed the two-level stabilized finite element method based on local Gauss integration. Readers can refer to [17,18,19,20] and the references cited to further understand the finite element iterative methods.

Recently, the FEM has been applied to solve the micropolar fluid equations. Ortega–Torres and Rojas–Medar [21] employed the fully discrete penalty finite element method to solve micropolar fluid equations, and proved optimal error estimates of linear velocity, pressure, and angular velocity. Nochetto et al. [22] proposed the first-order semi-implicit fully discrete finite element method. Jiang and Yang [23] proposed some projection methods and analyzed the stability and error estimates for the classical first-order projection scheme. Maimaiti and Liu [24] proposed first-order and second-order pressure-modified projection methods, and analysed the stability of time semi-discrete and fully discrete schemes.

According to the above literature summarization, the finite element method and three iterative methods have been successfully adopted to simulate Navier–Stokes equations, and some numerical achievements about the unsteady micropolar fluid equations are obtained. Numerical analysis and comparison of finite element iterative methods for the stationary micropolar fluid equation have not been reported in the open literature. It is well known that the stationary micropolar fluid equations are a strong coupled nonlinear system. This system contains two nonlinear terms, and velocity *u*, pressure *p* and angular velocity ω are coupled together. When the viscous effect in the micropolar fluid equations is weak, which means the nonlinear effect is strong, the main challenges of numerically solving it are how to construct efficient and stable iterative algorithms, rigorous theoretical analysis and application in practical problems. Based on the above difficulties, the three iterative methods are considered.

In this paper, three iterative methods are presented to simulate the stationary micropolar fluid equations for the different viscosity effect. The uniform stability and convergence of the proposed methods are also analysed. The main conclusions are reported in Theorems 5–9. In the numerical example section, we compared the effectiveness of the three iterative methods. Meanwhile, the classical benchmark problems and the bearing lubrication problem with inhomogeneous boundary conditions are simulated.

The rest of this paper is organized as follows. In Section 2, some necessary theories of Sobolev spaces are introduced. In Section 3, the stability and error estimation of the finite element discrete problem is provided. In Section 4, the main part of this paper, the stability and error estimation of the three iterative methods under different uniqueness conditions are analyzed and compared. In Section 5, some numerical tests are provided to illustrate the correctness of the theoretic analysis and contrast the effectiveness of the proposed methods. In Section 6, the main conclusions of this paper are presented.

## 2. Preliminaries

In this paper, we assume that Ω is a convex polygonal/polyhedral domain with Lipschitz continuous boundary in $R^{d}$, d=2 or 3. We consider the stationary micropolar fluid equations as follows [1,6]:

(1) $$\left\{\begin{array}{cc} -\nu_{0}\Delta u+\left(u\cdot \nabla \right)u+\nabla p=2\nu_{r}\nabla \times \omega +f, & \mathrm{in}\Omega ,\\ \nabla \cdot u=0, & \mathrm{in}\Omega ,\\ -c_{1}\Delta \omega +\left(u\cdot \nabla \right)\omega -c_{2}\nabla \nabla \cdot \omega +4\nu_{r}\omega =2\nu_{r}\nabla \times u+g, & \mathrm{in}\Omega , \end{array}\right.$$

where *u* is the velocity, *p* is the pressure, ω is the angular velocity, *f* and *g* are the external force terms, ν is the kinematic Newtonian viscosity, $\nu_{r}$ is the dynamic microrotation viscosity, and the positive constants $c_{a},c_{d},c_{0}$ are new viscosities connected with the asymmetry of the stress tensor, respectively. In order to simplify the calculation, we set $\nu_{0}=\nu +\nu_{r},c_{1}=c_{a}+c_{d},c_{2}=c_{0}+c_{a}-c_{d}>0$, and introduce the general definition of the curl operator as

$$a=\left(a_{1},a_{2},a_{3}\right),\;\;\;\nabla \times a=\left(\frac{\partial a_{3}}{\partial y}-\frac{\partial a_{2}}{\partial z},\frac{\partial a_{1}}{\partial z}-\frac{\partial a_{3}}{\partial x},\frac{\partial a_{2}}{\partial x}-\frac{\partial a_{1}}{\partial y}\right),\;\;\;\forall a\in R^{3}.$$

In particular, when d=2, let

$$u=\left(u_{1},u_{2},0\right),\;\;\nabla \times u=\left(0,0,\frac{\partial u_{2}}{\partial x}-\frac{\partial u_{1}}{\partial y}\right),\;\;\omega =\left(0,0,\omega_{3}\right),\;\;\nabla \times \omega =\left(\frac{\partial \omega_{3}}{\partial y},-\frac{\partial \omega_{3}}{\partial x},0\right).$$

For simplicity, let consider the following homogeneous boundary conditions:

(2) u=0   ,ω=0,   on∂Ω.

Let $W^{k,r}\left(\Omega \right)$ be the standard Sobolev spaces for all nonnegative integers *k* and *r* with norm ${∥\cdot ∥}_{k,r}$. As usual, the Hilbert space $H^{k}\left(\Omega \right)=W^{k,2}\left(\Omega \right)$ are equipped with norm ${∥v∥}_{k}$ when r=2. To obtain the weak formulation of (1)–(2), we introduce the following Sobolev spaces

$$\begin{array}{cc} X & =H_{0}^{1}{\left(\Omega \right)}^{d}=\left\{v\in H^{1}{\left(\Omega \right)}^{d}:v{|}_{\partial \Omega }=0\right\},\\ M & =L_{0}^{2}\left(\Omega \right)=\left\{q\in L^{2}\left(\Omega \right):\int_{\Omega }q\mathrm{dx}=0\right\},\\ V & =\{v\in X:\mathrm{div}v=0\mathrm{in}\Omega \}. \end{array}$$

For simplicity, we employ the product space W(Ω)=X×X with the usual graph norm ${∥\left(v,\psi \right)∥}_{i}={(∥v∥}_{i}^{2}+{∥\psi ∥}_{i}^{2}{)}^{1/2},i=0,1,2,$ for all (v,ψ)∈W(Ω). The space $H^{-1}{\left(\Omega \right)}^{d}$ denotes the dual of $H_{0}^{1}{\left(\Omega \right)}^{d}$ with the norm:

$${∥f∥}_{-1}=\underset{0\neq v\in H_{0}^{1}{\left(\Omega \right)}^{d}}{\sup}\frac{\langle f,v\rangle }{{∥v∥}_{1}},$$

where 〈·,·〉 denotes duality product between the function spaces $H^{1}{\left(\Omega \right)}^{d}$ and its dual.

For the purpose of the subsequent analysis, the following bilinear and trilinear forms are introduced:

$$\begin{array}{cc} & a\left(v,w\right)=\nu_{0}\left(\nabla v,\nabla w\right),\;\;\;\;c\left(v,\psi \right)=2\nu_{r}\left(\nabla \times v,\psi \right),\\ & \bar{a}\left(\psi ,\phi \right)=c_{1}\left(\nabla \psi ,\nabla \phi \right),\;\;\;\;\bar{k}\left(\psi ,\phi \right)=\left(\nabla \cdot \psi ,\nabla \cdot \phi \right),\\ & \bar{r}\left(\psi ,\phi \right)=4\nu_{r}\left(\psi ,\phi \right),\;\;\;\;d\left(\left(v,\psi \right),q\right)=\left(\nabla \cdot v,q\right),\\ & b\left(u,v,w\right)=\frac{1}{2}\left(u\cdot \nabla v,w\right)-\frac{1}{2}\left(u\cdot \nabla w,v\right),\\ & A\left(\left(v,\psi \right),\left(w,\phi \right)\right)=a\left(v,w\right)+\bar{a}\left(\psi ,\phi \right)-c\left(\psi ,w\right)-c\left(v,\phi \right)+\bar{k}\left(\psi ,\phi \right)+\bar{r}\left(\psi ,\phi \right),\\ & B((u,\omega ),(v,\psi ),(w,\phi ))=b(u,v,w)+b(v,\omega ,\phi ),\\ & \langle F,(v,\psi )\rangle =\langle f,v\rangle +\langle g,\psi \rangle . \end{array}$$

The following estimates about the trilinear form b(·,·,·) are classical [18,20,25].

(3) $$\begin{array}{cc} & b(u,v,w)=-b(u,w,v),\;\;\;\;\forall u,v,w\in X, \end{array}$$

(4) $$\begin{array}{c} \left|b\left(u,v,w\right)\right|\leq {N∥\nabla u∥}_{0}{∥\nabla v∥}_{0}{∥\nabla w∥}_{0},\;\;\;\forall u,v,w\in X, \end{array}$$

$$\begin{array}{c} \left|b\left(u,v,w\right)\right|\leq \frac{N}{2}{∥u∥}_{0}{(∥\nabla v∥}_{0}{∥w∥}_{L^{\infty }}+{∥v∥}_{L^{6}}{∥\nabla w∥}_{L^{3}}), \end{array}$$

(5) $$\begin{array}{c} \forall u\in L^{2}{\left(\Omega \right)}^{d},v\in X,w\in L^{\infty }{\left(\Omega \right)}^{d}\cap X, \end{array}$$

$$\begin{array}{c} \left|b\left(u,v,w\right)\right|\leq \frac{N}{2}{(∥u∥}_{L^{\infty }}{∥\nabla v∥}_{0}+{∥\nabla u∥}_{L^{3}}{∥v∥}_{L^{6}}{)∥w∥}_{0}, \end{array}$$

(6) $$\begin{array}{cc} & \forall u\in L^{\infty }{\left(\Omega \right)}^{d}\cap X,v\in X,w\in L^{2}{\left(\Omega \right)}^{d}, \end{array}$$

where *N* is a constant, $\gamma_{0}$ is a positive constant that may dependent on Ω. The following Sobolve spaces inequalities are used frequently

(7) $$\begin{array}{cc} & {∥v∥}_{0}\leq \gamma_{0}{∥\nabla v∥}_{0}{,∥v∥}_{L^{3}}\leq {C∥v∥}_{0}^{\frac{1}{2}}{∥\nabla v∥}_{0}^{\frac{1}{2}}{,∥v∥}_{L^{6}}\leq C{∥\nabla v∥}_{0},\;\;\;\;\forall v\in X, \end{array}$$

(8) $$\begin{array}{cc} & {∥v∥}_{L^{\infty }}\leq {C∥v∥}_{1}^{\frac{1}{2}}{∥v∥}_{2}^{\frac{1}{2}},\;\;\;\;\forall v\in H^{2}{\left(\Omega \right)}^{d}. \end{array}$$

With the above notations, the variational formulation of (1)–(2) reads: find ((u,ω),p)∈W×M such that

(9) $$\begin{array}{cc} & A((u,\omega ),(v,\psi ))+B((u,\omega ),(u,\omega ),(v,\psi ))-d((v,\psi ),p)+d((u,\omega ),q)\\ & \;\;\;\;=\langle F,(v,\psi )\rangle ,\;\;\;\;\forall ((v,\psi ),q)\in W\times M. \end{array}$$

According to the above estimates, 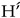 older inequality, Sobolev inequalities and the following inequalities

$${∥\nabla \times v∥}_{0}\leq \sqrt{2}{∥\nabla v∥}_{0},\;\;\;\;{∥\nabla \cdot v∥}_{0}\leq \sqrt{d}{∥\nabla v∥}_{0},$$

the following properties of A(·,·) and B(·,·,·) can be derived: ∀(u,ω),(v,ψ),(w,ϕ)∈W, there holds

(10) $$\begin{array}{cc} & A\left(\left(v,\psi \right),\left(w,\phi \right)\right)\leq C_{\max}{∥\left(v,\psi \right)∥}_{1}{∥\left(w,\phi \right)∥}_{1}, \end{array}$$

(11) $$\begin{array}{cc} & A\left(\left(v,\psi \right),\left(v,\psi \right)\right)\geq C_{\min}{∥\left(v,\psi \right)∥}_{1}^{2}, \end{array}$$

(12) $$\begin{array}{cc} & B((u,\omega ),(v,\psi ),(v,\psi ))=0, \end{array}$$

(13) $$\begin{array}{cc} & B\left(\left(u,\omega \right),\left(v,\psi \right),\left(w,\phi \right)\right)\leq {\lambda ∥\left(u,\omega \right)∥}_{1}{∥\left(v,\psi \right)∥}_{1}{∥\left(w,\phi \right)∥}_{1}, \end{array}$$

where $C_{\max}=\max\left\{\nu_{0}+2\sqrt{2}\nu_{r}\gamma_{0},c_{1}+c_{2}d+2\sqrt{2}\nu_{r}\gamma_{0}+4\nu_{r}\gamma_{0}^{2}\right\},C_{\min}=\min\left\{\nu_{0},c_{1}-c_{2}d\right\}$ and $\lambda =\sqrt{2}N$.

Note that the bilinear form d(·,·) is continuous on W×M and satisfies the classical inf–sup condition [6,18]:

(14) $$\underset{(0,0)\neq (v,\psi )\in W}{\sup}\frac{d((v,\psi ),q)}{{∥\left(v,\psi \right)∥}_{1}}\geq \beta_{0}{∥q∥}_{0},\;\;\;\;\forall q\in M.$$

Next, let us define the following norms:

$${∥F∥}_{-1}=\underset{(0,0)\neq (v,\psi )\in W}{\sup}\frac{\langle F,(v,\psi )\rangle }{{∥\left(v,\psi \right)∥}_{1}}{,\;\;\;\;\;\;∥F∥}_{i}={(∥f∥}_{i}^{2}+{∥g∥}_{i}^{2}{)}^{1/2},\;\;\;\;i=0,1,2.$$

For the sake of convenience in writing, we set

$$⫴\left(v,\psi \right){⫴}_{i}=C_{\min}{(∥v∥}_{i}^{2}+{∥\psi ∥}_{i}^{2}{)}^{1/2},\;\;\;\;\forall v,\psi \in H^{i}{\left(\Omega \right)}^{d}\cap X,\;\;\;\;i=0,1,2.$$

Łukaszewicz in [6] proved the existence and uniqueness of the weak solution of (1) and (2) by considering the auxiliary elliptical linear problem and adopting the Leray–Schauder principle. Next, we give a new proof of well-posedness by using the Banach fixed point theorem.

**Theorem** **1.** *If $\nu ,\nu_{r},c_{a},c_{d}$ and $c_{0}$ satisfy the uniqueness condition*

(15) $$0<\sigma =\frac{{\lambda ∥F∥}_{-1}}{C_{\min}^{2}}<1,$$

*then, there exists a unique solution to (9) and satisfies*

(16) $$⫴\left(u,\omega \right){⫴}_{1}\leq {∥F∥}_{-1}.$$

**Proof.**  Let us define the function space

Z={(u,ω)∈X×X:d((u,ω),q)=0,∀q∈M}.

For given (u,ω)∈Z, we consider the following linear problem:

(17) $$\begin{array}{cc} & A((w,\phi ),(v,\psi ))+B((u,\omega ),(w,\phi ),(v,\psi ))-d((v,\psi ),p)+d((w,\phi ),q)\\ & \;\;\;\;=\langle F,(v,\psi )\rangle ,\forall ((v,\psi ),q)\in W\times M. \end{array}$$

From the saddle-point theory [18], the problem (17) possesses a unique solution ((w,ϕ),p)∈W×M. Taking (v,ψ)=(w,ϕ) and q=p, using (11) and (12) we can drive the (16).

Next, let us prove the uniqueness condition (15). From the problem (17), we can define the mapping

Φ(u,ω)∈Z→(w,ϕ)∈Z,

such that $\Phi \left(u_{i},\omega_{i}\right)=\left(w_{i},\phi_{i}\right),i=1,2$, and $\left(w_{i},\phi_{i}\right)$ satisfy the following equation

(18) $$\begin{array}{cc} & A\left(\left(w_{i},\phi_{i}\right),\left(v,\psi \right)\right)+B\left(\left(u_{i},\omega_{i}\right),\left(w_{i},\phi_{i}\right),\left(v,\psi \right)\right)-d\left(\left(v,\psi \right),p\right)+d\left(\left(u_{i},\omega_{i}\right),q\right)\\ & =\langle F,(v,\psi )\rangle ,\forall ((v,\psi ),q)\in W\times M. \end{array}$$

Making a difference for i=1 and i=2 in (18) and taking $\left(v,\psi \right)=\left(w_{1}-w_{2},\phi_{1}-\phi_{2}\right)$ yields

(19) $$\begin{array}{cc} A & \left(\left(w_{1}-w_{2},\phi_{1}-\phi_{2}\right),\left(w_{1}-w_{2},\phi_{1}-\phi_{2}\right)\right)\\ & =-B(\left(u_{1}-u_{2},\omega_{1}-\omega_{2}\right),\left(w_{2},\phi_{2}\right),\left(w_{1}-w_{2},\phi_{1}-\phi_{2}\right)). \end{array}$$

By (11), (13) and (15), we have

(20) $$C_{\min}∥\Phi \left(u_{1},\omega_{1}\right)-\Phi \left(u_{2},\omega_{2}\right){∥}_{1}=C_{\min}∥\left(w_{1}-w_{2},\phi_{1}-\phi_{2}\right){∥}_{1}\leq \sigma {∥\left(u_{1}-u_{2},\omega_{1}-\omega_{2}\right)∥}_{1}.$$

According to the Banach fixed point theorem, we know that Φ has a fixed point in *Z*, which is the solution of problem (9). This completes the proof. □

**Theorem** **2.** *Suppose $f,g\in L^{2}{\left(\Omega \right)}^{d}$ and 0<σ<1, then the solution ((u,ω),p) of the problem (9) satisfies the following regularity*

(21) $$⫴\left(u,\omega \right){⫴}_{2}+{∥p∥}_{1}\leq C{∥F∥}_{0}.$$

**Proof.**  The proof is put in “Appendix A”. □

## 3. Finite Element Approximation

In this section, let $T_{h}$ be a regular and quasi-uniform partition of Ω into triangles for d=2 or tetrahedras for d=3, and $h=\max_{K\in T_{h}}h_{k}$ is a real positive parameter tending to 0. Next, we employ the following conforming finite element pairs to approximate the velocity, angular velocity and pressure.

$$\begin{array}{cc} X_{h} & =\{v_{h}\in C^{0}{\left(\bar{\Omega }\right)}^{d}\cap X:v_{h}{|}_{K}\in {\left(P_{1}\left(K\right)⊕\mathrm{span}\left\{\hat{b}\right\}\right)}^{d},\forall K\in T_{h}\},\\ M_{h} & =\{q_{h}\in C^{0}\left(\bar{\Omega }\right)\cap M:q_{h}{|}_{k}\in P_{1}\left(K\right),\forall K\in T_{h}\}, \end{array}$$

where $\hat{b}$ is the bubble function, and $P_{1}\left(K\right)$ denotes the space of polynomials of a degree equal to 1 on *K*. For convenience, let $W_{h}=X_{h}\times X_{h}$. In this case, $\left(W_{h},M_{h}\right)$ satisfies the following assumptions [6,17,18].

**Assumption** **1.** *There exists a positive constant*$\beta_{0}$*that only depends on* Ω *such that*

(22) $$\underset{\left(0,0\right)\neq \left(v,\psi \right)\in W_{h}}{\sup}\frac{d((v,\psi ),q)}{{∥\left(v,\psi \right)∥}_{1}}\geq \beta_{0}{∥q∥}_{0},\;\;\;\;\;\;\forall q\in M_{h}.$$

**Assumption** **2.** *There exists a mapping $R_{h}:H^{2}{\left(\Omega \right)}^{d}\cap V\to X_{h}$ satisfying*

(23) $$\left(\nabla \cdot \left(v-R_{h}v\right),q\right)=0\;\;\;\;,∥\nabla \left(v-R_{h}v\right){∥}_{0}\leq Ch{∥v∥}_{2},\;\;\;\;\forall v\in H^{2}{\left(\Omega \right)}^{d}\cap V,\;\;q\in M_{h},$$

*an $L^{2}\left(\Omega \right)$-orthogonal projection operator $\rho_{h}:M\to M_{h}$ satisfying*

(24) $$\left(\rho_{h}q,q_{h}\right)=\left(q,q_{h}\right),\;\;\;\;∥q-\rho_{h}{q∥}_{0}\leq Ch{∥q∥}_{1},\;\;\;\;\forall q\in H^{1}\left(\Omega \right)\cap M,q_{h}\in H^{1}\left(\Omega \right)\cap M_{h},$$

*and a mapping $Q_{h}:H^{2}{\left(\Omega \right)}^{d}\to X_{h}$ satisfying*

$$\left(\nabla Q_{h}\psi ,\nabla \phi \right)=\left(\nabla \psi ,\nabla \phi \right),\;\;\;\;\forall \phi \in W_{h},$$

(25) $$∥\psi -Q_{h}{\psi ∥}_{0}+h∥\psi -Q_{h}{\psi ∥}_{1}\leq Ch^{2}{∥\psi ∥}_{2},\;\;\;\;\forall \psi \in H^{2}{\left(\Omega \right)}^{d}\cap X.$$

With the above discrete spaces, the finite element approximation of (9) reads: find $\left(\left(u_{h},\omega_{h}\right),p_{h}\right)\in W_{h}\times M_{h}$ such that

(26) $$\begin{array}{cc} & A\left(\left(u_{h},\omega_{h}\right),\left(v,\psi \right)\right)+B\left(\left(u_{h},\omega_{h}\right),\left(u_{h},\omega_{h}\right),\left(v,\psi \right)\right)-d\left(\left(v,\psi \right),p_{h}\right)+d\left(\left(u_{h},\omega_{h}\right),q\right)\\ & \;\;\;\;=\langle F,\left(v,\psi \right)\rangle ,\forall \left(\left(v,\psi \right),q\right)\in W_{h}\times M_{h}. \end{array}$$

Similarly, let us define the discrete analogue of space *V* as

$$V_{h}=\left\{v\in X_{h}:d\left(\left(v,\psi \right),q\right)=0,\forall q\in M_{h},\psi \in W_{h}\right\},$$

and introduce the discrete Laplace operator $A_{h}=-\Delta_{h}$ as

$$\left(A_{h}v_{h},\psi_{h}\right)=\left(\nabla v_{h},\nabla \psi_{h}\right),\;\;\;\;\forall v_{h},\psi_{h}\in X_{h}.$$

Furthermore, we have the following discrete estimates [9,17]:

(27) $$∥\nabla v_{h}{∥}_{L^{3}}+∥v_{h}{∥}_{L^{\infty }}\leq C∥\nabla v_{h}{∥}_{0}^{\frac{1}{2}}∥A_{h}v_{h}{∥}_{0}^{\frac{1}{2}},\;\;\;\;∥\nabla v_{h}{∥}_{L^{6}}\leq C{∥A_{h}v_{h}∥}_{0},\;\;\;\;\;\;\forall v_{h}\in V_{h}.$$

Next, we prove the existence and uniqueness, stability and convergence of the discrete problem (26).

**Lemma** **1.** *The trilinear term B(·,·,·) satisfies the following estimate*

(28) $$\begin{array}{c} |B\left(\left(u_{h},\omega_{h}\right),\left(v_{h},\psi_{h}\right),\left(w_{h},\phi_{h}\right)\right)|+|B\left(\left(v_{h},\psi_{h}\right),\left(u_{h},\omega_{h}\right),\left(w_{h},\phi_{h}\right)\right)|\\ \;\;\;\;\leq C∥\left(u_{h},\omega_{h}\right){∥}_{1}^{1/2}∥\left(A_{h}u_{h},A_{h}\omega_{h}\right){∥}_{0}^{1/2}∥\left(v_{h},\psi_{h}\right){∥}_{1}{∥\left(w_{h},\phi_{h}\right)∥}_{0}. \end{array}$$

*for all*$\left(u_{h},\omega_{h}\right),\left(w_{h},\phi_{h}\right),\left(v_{h},\psi_{h}\right)\in W_{h}$.

**Proof.**  The estimate can be easily derived by (4)–(8) and 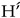
older inequality. □

**Theorem** **3.** *Suppose Assumption 1 and the uniqueness condition (15) hold, then the problem (26) exists a unique solution $\left(\left(u_{h},\omega_{h}\right),p_{h}\right)\in W_{h}\times M_{h}$ which satisfies*

(29) $$⫴\left(u_{h},\omega_{h}\right){⫴}_{1}\leq {∥F∥}_{-1},\;\;\;\;⫴\left(A_{h}u_{h},A_{h}\omega_{h}\right){⫴}_{0}\leq C{∥F∥}_{0}.$$

**Proof.**  The existence proof is obvious. Next, let us prove the stability and uniqueness.

For the first inequality, choosing $\left(v,\psi \right)=\left(u_{h},\omega_{h}\right)$ and $q=p_{h}$ in (26). Using (11) and (12) can easily get it.

For the second inequality, taking $\left(v,\psi \right)=\left(A_{h}u_{h},A_{h}\omega_{h}\right)$ and q=0 in (26), we have

(30) $$A\left(\left(u_{h},\omega_{h}\right),\left(A_{h}u_{h},A_{h}\omega_{h}\right)\right)=\langle F,\left(A_{h}u_{h},A_{h}\omega_{h}\right)\rangle -B\left(\left(u_{h},\omega_{h}\right),\left(u_{h},\omega_{h}\right),\left(A_{h}u_{h},A_{h}\omega_{h}\right)\right).$$

Using (28) and Young’s inequality yields

(31) $$\begin{array}{cc} ⫴\left(A_{h}u_{h},A_{h}\omega_{h}\right){⫴}_{0} & \leq {∥F∥}_{0}+C∥\left(u_{h},\omega_{h}\right){∥}_{1}^{\frac{3}{2}}{∥\left(A_{h}u_{h},A_{h}\omega_{h}\right)∥}_{0}^{\frac{1}{2}}\\ & \leq {∥F∥}_{0}+C⫴\left(u_{h},\omega_{h}\right){⫴}_{1}^{3}+\frac{1}{2}⫴\left(A_{h}u_{h},A_{h}\omega_{h}\right){⫴}_{0}. \end{array}$$

From (16) and (31), we can deduce (29). The proof is finished. □

**Theorem** **4.** *Suppose Assumptions 1 and 2 and the uniqueness condition (15) hold, we have the following error estimate*

(32) $$∥\left(u-u_{h},\omega -\omega_{h}\right){∥}_{0}+h(⫴\left(u-u_{h},\omega -\omega_{h}\right){⫴}_{1}+∥p-p_{h}{∥}_{0})\leq Ch^{2}.$$

**Proof.**  The proof is put in “Appendix B”. □

## 4. Three Iterative Methods

Considering the effect of different viscosity coefficients, the three iterative methods for micropolar fluid equations are proposed.

**Stokes iterative method:**

find $\left(\left(u_{h}^{n},\omega_{h}^{n}\right),p_{h}^{n}\right)\in W_{h}\times M_{h}$ such that for all $\left(\left(v,\psi \right),q\right)\in W_{h}\times M_{h}$

(33) $$\begin{array}{cc} & A\left(\left(u_{h}^{n},\omega_{h}^{n}\right),\left(v,\psi \right)\right)+B\left(\left(u_{h}^{n-1},\omega_{h}^{n-1}\right),\left(u_{h}^{n-1},\omega_{h}^{n-1}\right),\left(v,\psi \right)\right)-d\left(\left(v,\psi \right),p_{h}^{n}\right)\\ & \;\;\;\;+d\left(\left(u_{h}^{n},\omega_{h}^{n}\right),q\right)=\langle F,\left(v,\psi \right)\rangle . \end{array}$$

**Newton iterative method:**

find $\left(\left(u_{h}^{n},\omega_{h}^{n}\right),p_{h}^{n}\right)\in W_{h}\times M_{h}$ such that for all $\left(\left(v,\psi \right),q\right)\in W_{h}\times M_{h}$

(34) $$\begin{array}{cc} & A\left(\left(u_{h}^{n},\omega_{h}^{n}\right),\left(v,\psi \right)\right)+B\left(\left(u_{h}^{n-1},\omega_{h}^{n-1}\right),\left(u_{h}^{n},\omega_{h}^{n}\right),\left(v,\psi \right)\right)\\ & \;\;\;\;\;\;\;\;+B\left(\left(u_{h}^{n},\omega_{h}^{n}\right),\left(u_{h}^{n-1},\omega_{h}^{n-1}\right),\left(v,\psi \right)\right)-d\left(\left(v,\psi \right),p_{h}^{n}\right)+d\left(\left(u_{h}^{n},\omega_{h}^{n}\right),q\right)\\ & \;\;\;\;=B\left(\left(u_{h}^{n-1},\omega_{h}^{n-1}\right),\left(u_{h}^{n-1},\omega_{h}^{n-1}\right),\left(v,\psi \right)\right)+\langle F,\left(v,\psi \right)\rangle . \end{array}$$

**Oseen iterative method:**

find $\left(\left(u_{h}^{n},\omega_{h}^{n}\right),p_{h}^{n}\right)\in W_{h}\times M_{h}$ such that for all $\left(\left(v,\psi \right),q\right)\in W_{h}\times M_{h}$

(35) $$\begin{array}{cc} & A\left(\left(u_{h}^{n},\omega_{h}^{n}\right),\left(v,\psi \right)\right)+B\left(\left(u_{h}^{n-1},\omega_{h}^{n-1}\right),\left(u_{h}^{n},\omega_{h}^{n}\right),\left(v,\psi \right)\right)-d\left(\left(v,\psi \right),p_{h}^{n}\right)\\ & \;\;\;\;+d\left(\left(u_{h}^{n},\omega_{h}^{n}\right),q\right)=\langle F,\left(v,\psi \right)\rangle . \end{array}$$

The initial value $\left(\left(u_{h}^{0},\omega_{h}^{0}\right),p_{h}^{0}\right)$ is defined by:

(36) $$A\left(\left(u_{h}^{0},\omega_{h}^{0}\right),\left(v,\psi \right)\right)-d\left(\left(v,\psi \right),p_{h}^{0}\right)+d\left(\left(u_{h}^{0},\omega_{h}^{0}\right),q\right)=\langle F,\left(v,\psi \right)\rangle$$

for all $\left(\left(v,\psi \right),q\right)\in W_{h}\times M_{h}$.

Next, we establish the stability and error estimates of the three iterative methods. For simplicity, let $\left(e^{n},\xi^{n}\right)=\left(u_{h}-u_{h}^{n},\omega_{n}-\omega_{h}^{n}\right),\eta^{n}=p_{h}-p_{h}^{n}$.

**Lemma** **2.** *Under the conditions of Theorem 1, Assumptions 1 and 2, the initial value satisfies*

(37) $$⫴\left(u_{h}^{0},\omega_{h}^{0}\right){⫴}_{1}\leq {∥F∥}_{-1},\;\;\;\;⫴\left(A_{h}u_{h}^{0},A_{h}\omega_{h}^{0}\right){⫴}_{0}\leq {∥F∥}_{0},$$

*the initial errors $\left(e^{0},\xi^{0}\right)$ and $\eta^{0}$ satisfy the following bounds*

(38) $$⫴\left(e^{0},\xi^{0}\right){⫴}_{1}\leq {\sigma ∥F∥}_{-1},\;\;\;\;∥\eta^{0}{∥}_{0}\leq C\sigma {∥F∥}_{-1}.$$

**Proof.**  Let $\left(v,\psi \right)=\left(u_{h}^{0},\omega_{h}^{0}\right)$, $q=p_{h}^{0}$ in (36) and applying (11), the first term of (37) can be deduced. Similarly, choosing $\left(v,\psi \right)=\left(A_{h}u_{h}^{0},A_{h}\omega_{h}^{0}\right)$ and q=0, the second term of (37) can be derived.

Next, let estimate the initial errors bounds (38). Subtracting (36) from (26), we have

(39) $$A\left(\left(e^{0},\xi^{0}\right),\left(v,\psi \right)\right)-d\left(\left(v,\psi \right),\eta^{0}\right)+d\left(\left(e^{0},\xi^{0}\right),q\right)+B\left(\left(u_{h},\omega_{h}\right),\left(u_{h},\omega_{h}\right),\left(v,\psi \right)\right)=0.$$

Taking $\left(v,\psi \right)=\left(e^{0},\xi^{0}\right),q=\eta^{0}$ in (39) and using (11), (13), (15) and (16), we obtain

(40) $$⫴\left(e^{0},\xi^{0}\right){⫴}_{1}\leq \frac{\lambda }{C_{\min}^{2}}⫴\left(u_{h},\omega_{h}\right){⫴}_{1}^{2}\leq \sigma {∥F∥}_{-1}.$$

Then, using the discrete inf-sup condition (22) gives that

(41) $$\beta_{0}∥\eta^{0}{∥}_{0}\leq \frac{C_{\max}}{C_{\min}}⫴\left(e^{0},\xi^{0}\right){⫴}_{1}+\frac{\lambda }{C_{\min}^{2}}⫴\left(u_{h},\omega_{h}\right){⫴}_{1}^{2}\leq C\sigma {∥F∥}_{-1}.$$

The proof is completed. □

**Theorem** **5.** *Under the conditions of Theorem 1, Assumptions 1 and 2, if*

(42) $$0<\sigma <\frac{2}{5},$$

*then $\left(u_{h}^{m},\omega_{h}^{m}\right)$ defined by the Stokes iterative method satisfies*

(43) $$⫴\left(u_{h}^{m},\omega_{h}^{m}\right){⫴}_{1}\leq \frac{6}{5}{∥F∥}_{-1},\;\;\;\;⫴\left(A_{h}u_{h}^{m},A_{h}\omega_{h}^{m}\right){⫴}_{0}\leq C{∥F∥}_{0},$$

*and $\left(e^{m},\xi^{m}\right),\eta^{m}$ satisfy the following bounds*

(44) $$⫴\left(e^{m},\xi^{m}\right){⫴}_{1}\leq {\left(\frac{11}{5}\sigma \right)}^{m}\frac{2}{5}{∥F∥}_{-1},∥\eta^{m}{∥}_{0}\leq C{\left(\frac{11}{5}\sigma \right)}^{m}\frac{2}{5}{∥F∥}_{-1}$$

*for all*m≥0.

**Proof.**  Firstly, we give the stability estimate. By Lemma 4.1, it can be checked that (43) is valid for m=0. Let n=1 in (33) and subtract (36) from (33) with $\left(v,\psi \right)=\left(u_{h}^{1}-u_{h}^{0},\omega_{h}^{1}-\omega_{h}^{0}\right),q=p_{h}^{1}-p_{h}^{0}$, we have

(45) $$A\left(\left(u_{h}^{1}-u_{h}^{0},\omega_{h}^{1}-\omega_{h}^{0}\right),\left(u_{h}^{1}-u_{h}^{0},\omega_{h}^{1}-\omega_{h}^{0}\right)\right)=-B\left(\left(u_{h}^{0},\omega_{h}^{0}\right),\left(u_{h}^{0},\omega_{h}^{0}\right),\left(u_{h}^{1}-u_{h}^{0},\omega_{h}^{1}-\omega_{h}^{0}\right)\right).$$

Using (11), (13) and (42), we obtain

(46) $$⫴\left(u_{h}^{1}-u_{h}^{0},\omega_{h}^{1}-\omega_{h}^{0}\right){⫴}_{1}\leq \frac{\lambda }{C_{\min}^{2}}⫴\left(u_{h}^{0},\omega_{h}^{0}\right){⫴}_{1}^{2}\leq {\sigma ∥F∥}_{-1}\leq \frac{2}{5}{∥F∥}_{-1}.$$

Then, let n=1 and taking $\left(v,\psi \right)=\left(u_{h}^{1},\omega_{h}^{1}\right),q=p_{h}^{1}$ in (33), we have

(47) $$A\left(\left(u_{h}^{1},\omega_{h}^{1}\right),\left(u_{h}^{1},\omega_{h}^{1}\right)\right)-B\left(\left(u_{h}^{0},\omega_{h}^{0}\right),\left(u_{h}^{1}-u_{h}^{0},\omega_{h}^{1}-\omega_{h}^{0}\right),\left(u_{h}^{1},\omega_{h}^{1}\right)\right)=\langle F,\left(u_{h}^{1},\omega_{h}^{1}\right)\rangle .$$

From (11), (13) and (46), we derive that

(48) $$\begin{array}{cc} ⫴\left(u_{h}^{1},\omega_{h}^{1}\right){⫴}_{1} & \leq {∥F∥}_{-1}+\frac{\lambda }{C_{\min}^{2}}⫴\left(u_{h}^{0},\omega_{h}^{0}\right){⫴}_{1}⫴\left(u_{h}^{1}-u_{h}^{0},\omega_{h}^{1}-\omega_{h}^{0}\right){⫴}_{1}\\ & \leq \left(1+\sigma^{2}\right){∥F∥}_{-1}\leq \frac{6}{5}{∥F∥}_{-1}. \end{array}$$

Suppose that (43) holds for m=1,···,n−1. We now turn to prove that it is right for m=n. Two adjacent iterative steps are subtracted, and taking $\left(v,\psi \right)=\left(u_{h}^{n}-u_{h}^{n-1},\omega_{h}^{n}-\omega_{h}^{n-1}\right),q=p_{h}^{n}-p_{h}^{n-1}$ in (33), we deduce that

(49) $$\begin{array}{cc} & A(\left(u_{h}^{n}-u_{h}^{n-1},\omega_{h}^{n}-\omega_{h}^{n-1}\right),\left(u_{h}^{n}-u_{h}^{n-1},\omega_{h}^{n}-\omega_{h}^{n-1}\right))\\ & \;\;\;\;\;\;\;\;+B(\left(u_{h}^{n-1},\omega_{h}^{n-1}\right),\left(u_{h}^{n-1}-u_{h}^{n-2},\omega_{h}^{n-1}-\omega_{h}^{n-2}\right),\left(u_{h}^{n}-u_{h}^{n-1},\omega_{h}^{n}-\omega_{h}^{n-1}\right))\\ & \;\;\;\;=-B(\left(u_{h}^{n-1}-u_{h}^{n-2},\omega_{h}^{n-1}-\omega_{h}^{n-2}\right),\left(u_{h}^{n-2},\omega_{h}^{n-2}\right),\left(u_{h}^{n}-u_{h}^{n-1},\omega_{h}^{n}-\omega_{h}^{n-1}\right)). \end{array}$$

Then, applying (11), (13), (42) and (46), we obtain

(50) $$\begin{array}{cc} & ⫴\left(u_{h}^{n}-u_{h}^{n-1},\omega_{h}^{n}-\omega_{h}^{n-1}\right){⫴}_{1}\\ & \;\;\;\leq \frac{\lambda }{C_{\min}^{2}}\left(⫴\left(u_{h}^{n-1},\omega_{h}^{n-1}\right){⫴}_{1}+⫴\left(u_{h}^{n-2},\omega_{h}^{n-2}\right){⫴}_{1}\right)⫴\left(u_{h}^{n-1}-u_{h}^{n-2},\omega_{h}^{n-1}-\omega_{h}^{n-2}\right){⫴}_{1}\\ & \;\;\;\leq \frac{24}{25}⫴\left(u_{h}^{n-1}-u_{h}^{n-2},\omega_{h}^{n-1}-\omega_{h}^{n-2}\right){⫴}_{1}\leq {\left(\frac{24}{25}\right)}^{n-1}⫴\left(u_{h}^{1}-u_{h}^{0},\omega_{h}^{1}-\omega_{h}^{0}\right){⫴}_{1}\\ & \;\;\;\leq \frac{2}{5}{∥F∥}_{-1}. \end{array}$$

In terms of (33), taking $\left(v,\psi \right)=\left(u_{h}^{n},\omega_{h}^{n}\right)$ and $q=p_{h}^{n}$ leads to

(51) $$A\left(\left(u_{h}^{n},\omega_{h}^{n}\right),\left(u_{h}^{n},\omega_{h}^{n}\right)\right)-B\left(\left(u_{h}^{n-1},\omega_{h}^{n-1}\right),\left(u_{h}^{n}-u_{h}^{n-1},\omega_{h}^{n}-\omega_{h}^{n-1}\right),\left(u_{h}^{n},\omega_{h}^{n}\right)\right)=\langle F,\left(u_{h}^{n},\omega_{h}^{n}\right)\rangle .$$

Together with (11), (13), (42) and (50), we have

(52) $$\begin{array}{cc} ⫴\left(u_{h}^{n},\omega_{h}^{n}\right){⫴}_{1} & \leq \frac{\lambda }{C_{\min}^{2}}⫴\left(u_{h}^{n-1},\omega_{h}^{n-1}\right){⫴}_{1}⫴\left(u_{h}^{n}-u_{h}^{n-1},\omega_{h}^{n}-\omega_{h}^{n-1}\right){⫴}_{1}+{∥F∥}_{-1}\\ & \leq \left(1+\frac{12}{25}\sigma \right){∥F∥}_{-1}\leq \frac{6}{5}{∥F∥}_{-1}. \end{array}$$

Next, taking $\left(v,\psi \right)=\left(A_{h}u_{h}^{n},A_{h}\omega_{h}^{n}\right),q=0$ in (33) and using Lemma 4.1, we have

(53) $$⫴\left(A_{h}u_{h}^{n},A_{h}\omega_{h}^{n}\right){⫴}_{0}\leq {∥F∥}_{0}+C∥\left(u_{h}^{n-1},\omega_{h}^{n-1}\right){∥}_{1}^{\frac{3}{2}}{∥\left(A_{h}u_{h}^{n},A_{h}\omega_{h}^{n}\right)∥}_{0}^{\frac{1}{2}}.$$

If $∥\left(A_{h}u_{h}^{n},A_{h}\omega_{h}^{n}\right){∥}_{0}\leq {∥\left(A_{h}u_{h}^{n-1},A_{h}\omega_{h}^{n-1}\right)∥}_{0}$, combining (42) and (52) yields

(54) $$⫴\left(A_{h}u_{h}^{n},A_{h}\omega_{h}^{n}\right){⫴}_{0}\leq C{∥F∥}_{0}.$$

Furthermore, we assume $∥\left(A_{h}u_{h}^{n-1},A_{h}\omega_{h}^{n-1}\right){∥}_{0}\leq {∥\left(A_{h}u_{h}^{n},A_{h}\omega_{h}^{n}\right)∥}_{0}$, from (53) and Young’s inequality that

(55) $${⫴(A_{h}u_{h}^{n},A_{h}\omega_{h}^{n})⫴}_{0}\leq 2{∥F∥}_{0}+C\sigma^{2}{∥F∥}_{0}\leq C{∥F∥}_{0}.$$

Finally, we estimate the bounds of $\left(e^{m},\xi^{m}\right)$ and $\eta^{m}$. Subtracting (33) from (26), we have

(56) $$\begin{array}{cc} & A\left(\left(e^{n},\xi^{n}\right),\left(v,\psi \right)\right)-d\left(\left(v,\psi \right),\eta^{n}\right)+d\left(\left(e^{n},\xi^{n}\right),q\right)+B\left(\left(e^{n-1},\xi^{n-1}\right),\left(u_{h},\omega_{h}\right),\left(v,\psi \right)\right)\\ & \;\;\;\;+B(\left(u_{h}^{n-1},\omega_{h}^{n-1}\right),\left(e^{n-1},\xi^{n-1}\right),\left(v,\psi \right))=0. \end{array}$$

It is obvious that (44) holds for m=0. Suppose that (44) holds for m=1,···,n−1. Let us prove it valid for m=n. Taking $\left(v,\psi \right)=\left(e^{n},\xi^{n}\right),q=\eta^{n}$ in (56) and using (11)–(13), (16), (42) and (52) lead to

(57) $$\begin{array}{cc} ⫴\left(e^{n},\xi^{n}\right){⫴}_{1} & \leq \frac{\lambda }{C_{\min}^{2}}\left(⫴\left(u_{h},\omega_{h}\right){⫴}_{1}+⫴\left(u_{h}^{n-1},\omega_{h}^{n-1}\right){⫴}_{1}\right)⫴\left(e^{n-1},\xi^{n-1}\right){⫴}_{1}\\ & \leq \frac{11}{5}\sigma ⫴\left(e^{n-1},\xi^{n-1}\right){⫴}_{1}\leq {\left(\frac{11}{5}\sigma \right)}^{n}⫴\left(e^{0},\xi^{0}\right){⫴}_{1}\\ & \leq {\left(\frac{11}{5}\sigma \right)}^{n}\frac{2}{5}{∥F∥}_{-1}. \end{array}$$

In terms of (56), using the discrete inf-sup condition (22), we obtain

(58) $$\begin{array}{cc} \beta_{0}{∥\eta^{n}∥}_{0} & \leq \frac{C_{\max}}{C_{\min}}⫴\left(e^{n},\xi^{n}\right){⫴}_{1}+\frac{\lambda }{C_{\min}^{2}}\left(⫴\left(u_{h}^{n-1},\omega_{h}^{n-1}\right){⫴}_{1}+⫴\left(u_{h},\omega_{h}\right){⫴}_{1}\right)⫴\left(e^{n-1},\xi^{n-1}\right){⫴}_{1}\\ & \leq \frac{C_{\max}}{C_{\min}}{\left(\frac{11}{5}\sigma \right)}^{n}\frac{2}{5}{∥F∥}_{-1}+{\left(\frac{11}{5}\sigma \right)}^{n}\frac{2}{5}{∥F∥}_{-1}\\ & \leq C{\left(\frac{11}{5}\sigma \right)}^{n}\frac{2}{5}{∥F∥}_{-1}. \end{array}$$

The proof is finished. □

**Theorem** **6.** *Under the conditions of Theorem 1, Assumptions 1 and 2, if*

(59) $$0<\sigma <\frac{5}{11},$$

*then $\left(u_{h}^{m},\omega_{h}^{m}\right)$ defined by the Newton iterative method satisfies*

(60) $$⫴\left(u_{h}^{m},\omega_{h}^{m}\right){⫴}_{1}\leq \frac{4}{3}{∥F∥}_{-1},\;\;\;\;⫴\left(A_{h}u_{h}^{m},A_{h}\omega_{h}^{m}\right){⫴}_{0}\leq C{∥F∥}_{0},$$

*and $\left(e^{m},\xi^{m}\right),\eta^{m}$ satisfy the following bounds*

(61) $$⫴\left(e^{m},\xi^{m}\right){⫴}_{1}\leq {\left(\frac{15}{13}\sigma \right)}^{2^{m}-1}\frac{5}{11}{∥F∥}_{-1},\;\;\;\;∥\eta^{m}{∥}_{0}\leq C{\left(\frac{15}{13}\sigma \right)}^{2^{m}-1}\frac{5}{11}{∥F∥}_{-1}$$

*for all m≥0.*

**Proof.**  By Lemma 4.1, we know that the (60) holds for m=0. Setting n=1 in (34) and subtracting (36) from (34) with $\left(v,\psi \right)=\left(u_{h}^{1}-u_{h}^{0},\omega_{h}^{1}-\omega_{h}^{0}\right)$, we obtain

(62) $$\begin{array}{cc} & A(\left(u_{h}^{1}-u_{h}^{0},\omega_{h}^{1}-\omega_{h}^{0}\right),\left(u_{h}^{1}-u_{h}^{0},\omega_{h}^{1}-\omega_{h}^{0}\right))\\ & \;\;\;\;\;\;\;\;+B(\left(u_{h}^{1}-u_{h}^{0},\omega_{h}^{1}-\omega_{h}^{0}\right),\left(u_{h}^{0},\omega_{h}^{0}\right),\left(u_{h}^{1}-u_{h}^{0},\omega_{h}^{1}-\omega_{h}^{0}\right))\\ & \;\;\;\;=-B(\left(u_{h}^{0},\omega_{h}^{0}\right),\left(u_{h}^{0},\omega_{h}^{0}\right),\left(u_{h}^{1}-u_{h}^{0},\omega_{h}^{1}-\omega_{h}^{0}\right)). \end{array}$$

Combining (11), (13) and (59), we derive that

(63) $$\begin{array}{cc} \frac{6}{11}⫴\left(u_{h}^{1}-u_{h}^{0},\omega_{h}^{1}-\omega_{h}^{0}\right){⫴}_{1} & \leq \left(1-\sigma \right)⫴\left(u_{h}^{1}-u_{h}^{0},\omega_{h}^{1}-\omega_{h}^{0}\right){⫴}_{1}\\ & \leq \frac{\lambda }{C_{\min}^{2}}⫴\left(u_{h}^{0},\omega_{h}^{0}\right){⫴}_{1}^{2}\leq {\sigma ∥F∥}_{-1}\leq \frac{5}{11}{∥F∥}_{-1}. \end{array}$$

Taking $\left(v,\psi \right)=\left(u_{h}^{1},\omega_{h}^{1}\right)$ and $q=p_{h}^{1}$ in (34), we have

(64) $$A\left(\left(u_{h}^{1},\omega_{h}^{1}\right),\left(u_{h}^{1},\omega_{h}^{1}\right)\right)-B\left(\left(u_{h}^{1}-u_{h}^{0},\omega_{h}^{1}-\omega_{h}^{0}\right),\left(u_{h}^{1}-u_{h}^{0},\omega_{h}^{1}-\omega_{h}^{0}\right),\left(u_{h}^{1},\omega_{h}^{1}\right)\right)=\langle F,\left(u_{h}^{1},\omega_{h}^{1}\right)\rangle .$$

Using (11), (13), (59) and (63), we obtain

(65) $$⫴\left(u_{h}^{1},\omega_{h}^{1}\right){⫴}_{1}\leq {∥F∥}_{-1}+\frac{\lambda }{C_{\min}^{2}}⫴\left(u_{h}^{1}-u_{h}^{0},\omega_{h}^{1}-\omega_{h}^{0}\right){⫴}_{1}^{2}\leq \left(1+\frac{25}{36}\sigma \right){∥F∥}_{-1}\leq \frac{4}{3}{∥F∥}_{-1}.$$

Suppose that (60) holds for m=1,···,n−1, now we prove that it is right for m=n. Let $\left(v,\psi \right)=\left(u_{h}^{n}-u_{h}^{n-1},\omega_{h}^{n}-\omega_{h}^{n-1}\right)$ in (34), we have

(66) $$\begin{array}{cc} & A(\left(u_{h}^{n}-u_{h}^{n-1},\omega_{h}^{n}-\omega_{h}^{n-1}\right),\left(u_{h}^{n}-u_{h}^{n-1},\omega_{h}^{n}-\omega_{h}^{n-1}\right))\\ & \;\;\;\;\;\;\;\;+B(\left(u_{h}^{n}-u_{h}^{n-1},\omega_{h}^{n}-\omega_{h}^{n-1}\right),\left(u_{h}^{n-1},\omega_{h}^{n-1}\right),\left(u_{h}^{n}-u_{h}^{n-1},\omega_{h}^{n}-\omega_{h}^{n-1}\right))\\ & \;\;\;\;=-B(\left(u_{h}^{n-1}-u_{h}^{n-2},\omega_{h}^{n-1}-\omega_{h}^{n-2}\right),\left(u_{h}^{n-1}-u_{h}^{n-2},\omega_{h}^{n-1}-\omega_{h}^{n-2}\right),\left(u_{h}^{n}-u_{h}^{n-1},\omega_{h}^{n}-\omega_{h}^{n-1}\right)). \end{array}$$

From (11), (13) and (59), we have

(67) $$\begin{array}{cc} \frac{13}{33}⫴\left(u_{h}^{n}-u_{h}^{n-1},\omega_{h}^{n}-\omega_{h}^{n-1}\right){⫴}_{1} & \leq \left(1-\frac{4}{3}\sigma \right)⫴\left(u_{h}^{n}-u_{h}^{n-1},\omega_{h}^{n}-\omega_{h}^{n-1}\right){⫴}_{1}\\ & \leq \frac{\lambda }{C_{\min}^{2}}⫴\left(u_{h}^{n-1}-u_{h}^{n-2},\omega_{h}^{n-1}-\omega_{h}^{n-2}\right){⫴}_{1}^{2}. \end{array}$$

From (59), (63) and (67), the following identity can be derived

(68) $$\begin{array}{cc} & ⫴\left(u_{h}^{n}-u_{h}^{n-1},\omega_{h}^{n}-\omega_{h}^{n-1}\right){⫴}_{1}\\ & \;\;\;\leq \frac{33}{13}\frac{\lambda }{C_{\min}^{2}}⫴\left(u_{h}^{n-1}-u_{h}^{n-2},\omega_{h}^{n-1}-\omega_{h}^{n-2}\right){⫴}_{1}^{2}\\ & \;\;\;\leq {\left(\frac{33}{13}\frac{\lambda }{C_{\min}}⫴\left(u_{h}^{1}-u_{h}^{0},\omega_{h}^{1}-\omega_{h}^{0}\right){⫴}_{1}\right)}^{2^{n-1}-1}⫴\left(u_{h}^{1}-u_{h}^{0},\omega_{h}^{1}-\omega_{h}^{0}\right){⫴}_{1}\\ & \;\;\;\leq {\left(\frac{25}{26}\right)}^{2^{n-1}-1}\frac{5}{6}{∥F∥}_{-1}\leq \frac{5}{6}{∥F∥}_{-1}. \end{array}$$

Taking $\left(v,\psi \right)=\left(u_{h}^{n},\omega_{h}^{n}\right)$ and $q=p_{h}^{n}$ in (34), we have

(69) $$\begin{array}{cc} & A\left(\left(u_{h}^{n},\omega_{h}^{n}\right),\left(u_{h}^{n},\omega_{h}^{n}\right)\right)-B\left(\left(u_{h}^{n}-u_{h}^{n-1},\omega_{h}^{n}-\omega_{h}^{n-1}\right),\left(u_{h}^{n}-u_{h}^{n-1},\omega_{h}^{n}-\omega_{h}^{n-1}\right),\left(u_{h}^{n},\omega_{h}^{n}\right)\right)\\ & \;\;\;\;=\langle F,\left(u_{h}^{n},\omega_{h}^{n}\right)\rangle . \end{array}$$

From (11), (59) and (69), we deduce that

(70) $$⫴\left(u_{h}^{n},\omega_{h}^{n}\right){⫴}_{1}\leq \left(1+\frac{25}{36}\sigma \right){∥F∥}_{-1}\leq \frac{4}{3}{∥F∥}_{-1}.$$

Now, we prove the second term of (60) holds for m=n. Assuming it is correct for m=0,1,···,n−1. Taking $\left(v,\psi \right)=\left(A_{h}u_{h}^{n},A_{h}\omega_{h}^{n}\right),q=0$ in (34) and combining Lemma 4.1 and Young’s inequality, we have

(71) $$\begin{array}{cc} ⫴\left(A_{h}u_{h}^{n},A_{h}\omega_{h}^{n}\right){⫴}_{0} & \leq {∥F∥}_{0}+C∥\left(u_{h}^{n-1},\omega_{h}^{n-1}\right){∥}_{1}^{\frac{3}{2}}{∥\left(A_{h}u_{h}^{n-1},A_{h}\omega_{h}^{n-1}\right)∥}_{0}^{\frac{1}{2}}\\ & \;\;\;+C∥\left(u_{h}^{n-1},\omega_{h}^{n-1}\right){∥}_{1}∥\left(u_{h}^{n},\omega_{h}^{n}\right){∥}_{1}^{\frac{1}{2}}{∥\left(A_{h}u_{h}^{n},A_{h}\omega_{h}^{n}\right)∥}_{0}^{\frac{1}{2}}\\ & \leq {∥F∥}_{0}+C∥\left(u_{h}^{n-1},\omega_{h}^{n-1}\right){∥}_{1}^{3}+C∥\left(u_{h}^{n-1},\omega_{h}^{n-1}\right){∥}_{1}^{2}{∥\left(u_{h}^{n},\omega_{h}^{n}\right)∥}_{1}\\ & \;\;\;+\frac{1}{4}⫴\left(A_{h}u_{h}^{n-1},A_{h}\omega_{h}^{n-1}\right){⫴}_{0}+\frac{1}{4}⫴\left(A_{h}u_{h}^{n},A_{h}\omega_{h}^{n}\right){⫴}_{0}. \end{array}$$

Applying the similar technique used in Theorem 5 and some simple calculations, we can obtain the second term of (60).

Next, we give the estimation of error bounds. Subtracting (34) from (28), we have

(72) $$\begin{array}{cc} & A\left(\left(e^{n},\xi^{n}\right),\left(v,\psi \right)\right)-d\left(\left(v,\psi \right),\eta^{n}\right)+d\left(\left(e^{n},\xi^{n}\right),q\right)\\ & \;\;\;\;+B\left(\left(u_{h}^{n-1},\omega_{h}^{n-1}\right),\left(e^{n},\xi^{n}\right),\left(v,\psi \right)\right)+B\left(\left(e^{n},\xi^{n}\right),\left(u_{h}^{n-1},\omega_{h}^{n-1}\right),\left(v,\psi \right)\right)\\ & \;\;\;\;+B(\left(e^{n-1},\xi^{n-1}\right),\left(e^{n-1},\xi^{n-1}\right),\left(v,\psi \right))=0. \end{array}$$

The Equation (38) shows the first term (61) holds for m=0. Suppose that it holds for m=n−1. Taking $m=n,\left(v,\psi \right)=\left(e^{n},\xi^{n}\right)$ and $q=\eta^{n}$ in (72), using (11), (13), (16), (59) and (70), we conclude that

(73) $$\frac{13}{33}⫴\left(e^{n},\xi^{n}\right){⫴}_{1}\leq \left(1-\frac{4}{3}\sigma \right)⫴\left(e^{n},\xi^{n}\right){⫴}_{1}\leq \frac{\lambda }{C_{\min}^{2}}⫴\left(e^{n-1},\xi^{n-1}\right){⫴}_{1}^{2}.$$

which yields that

(74) $$\begin{array}{cc} ⫴\left(e^{n},\xi^{n}\right){⫴}_{1} & \leq \frac{33}{13}\frac{\lambda }{C_{\min}^{2}}⫴\left(e^{n-1},\xi^{n-1}\right){⫴}_{1}^{2}\leq {\left(\frac{33}{13}\frac{\lambda }{C_{\min}^{2}}\right)}^{2^{n}-1}⫴\left(e^{0},\xi^{0}\right){⫴}_{1}^{2^{n}}\\ & \leq {\left(\frac{15}{13}\sigma \right)}^{2^{n}-1}\frac{5}{11}{∥F∥}_{-1}. \end{array}$$

In terms of (72), using the discrete inf–sup condition (22) arrives that

(75) $$\begin{array}{cc} \beta_{0}{∥\eta^{n}∥}_{0} & \leq \frac{C_{\max}}{C_{\min}}⫴\left(e^{n},\xi^{n}\right){⫴}_{1}+\frac{\lambda }{C_{\min}^{2}}\left(⫴\left(e^{n-1},\xi^{n-1}\right){⫴}_{1}^{2}+2⫴\left(u_{h}^{n-1},\omega_{h}^{n-1}\right){⫴}_{1}⫴\left(e^{n},\xi^{n}\right){⫴}_{1}\right)\\ & \leq C{\left(\frac{15}{13}\sigma \right)}^{2^{n}-1}\frac{5}{11}{∥F∥}_{-1}+{\left(\frac{15}{13}\sigma \right)}^{2^{n}-1}\frac{5}{11}{∥F∥}_{-1}\\ & \leq C{\left(\frac{15}{13}\sigma \right)}^{2^{n}-1}\frac{5}{11}{∥F∥}_{-1}. \end{array}$$

The proof is finished. □

**Theorem** **7.** *Under the conditions of Theorem 1, Assumptions 1 and 2, if*

(76) 0<σ<1,

*then $\left(u_{h}^{m},\omega_{h}^{m}\right)$ defined by the Oseen iterative method satisfies*

(77) $$⫴\left(u_{h}^{m},\omega_{h}^{m}\right){⫴}_{1}\leq {∥F∥}_{-1},\;\;\;\;⫴\left(A_{h}u_{h}^{m},A_{h}\omega_{h}^{m}\right){⫴}_{0}\leq C{∥F∥}_{0},$$

$\left(e^{n},\xi^{n}\right)$ *and* $\eta^{n}$ *satisfy the following bounds*

(78) $$⫴\left(e^{m},\xi^{m}\right){⫴}_{1}\leq \sigma^{m}{∥F∥}_{-1},∥\eta^{m}{∥}_{0}\leq C\sigma^{m}{∥F∥}_{-1}$$

*for all m≥0.*

**Proof.**  We can easily deduce that (77) holds when m=0. Taking $\left(v,\psi \right)=\left(u_{h}^{n},\omega_{h}^{n}\right),q=p_{h}^{n}$ in (35), and applying (11), (12) and (15), we obtain the first term of (77).

Then, setting $\left(v,\psi \right)=\left(A_{h}u_{h}^{n},A_{h}\omega_{h}^{n}\right)$ and q=0 in (35), we obtain

(79) $$\begin{array}{cc} ⫴\left(A_{h}u_{h}^{n},A_{h}\omega_{h}^{n}\right){⫴}_{1} & \leq {∥F∥}_{0}+C∥\left(u_{h}^{n-1},\omega_{h}^{n-1}\right){∥}_{1}∥\left(u_{h}^{n},\omega_{h}^{n}\right){∥}_{1}^{\frac{1}{2}}{∥\left(A_{h}u_{h}^{n},A_{h}\omega_{h}^{n}\right)∥}_{0}^{\frac{1}{2}}\\ & \leq {∥F∥}_{0}+C∥\left(u_{h}^{n-1},\omega_{h}^{n-1}\right){∥}_{1}^{2}{∥\left(u_{h}^{n},\omega_{h}^{n}\right)∥}_{1}+\frac{1}{2}⫴\left(A_{h}u_{h}^{n},A_{h}\omega_{h}^{n}\right){⫴}_{1}. \end{array}$$

By using the same technique of Theorem 5, we get the second term of (77).

Next, we prove the error bounds. Subtracting (35) from (26) yields

(80) $$\begin{array}{cc} & A\left(\left(e^{n},\xi^{n}\right),\left(v,\psi \right)\right)-d\left(\left(v,\psi \right),\eta^{n}\right)+d\left(\left(e^{n},\xi^{n}\right),q\right)\\ & \;\;\;\;+B\left(\left(e^{n-1},\xi^{n-1}\right),\left(u_{h},\omega_{h}\right),\left(v,\psi \right)\right)+B\left(\left(u_{h}^{n-1},\omega_{h}^{n-1}\right),\left(e^{n},\xi^{n}\right),\left(v,\psi \right)\right)=0. \end{array}$$

Obviously, the Equation (78) holds for m=0. Taking $m=n,\left(v,\psi \right)=\left(e^{n},\xi^{n}\right),q=\eta^{n}$ in (80) and applying (11), (13), (29) and (76), we obtain

(81) $$\begin{array}{cc} ⫴\left(e^{n},\xi^{n}\right){⫴}_{1} & \leq \frac{\lambda }{C_{\min}^{2}}⫴\left(u_{h},\omega_{h}\right){⫴}_{1}⫴\left(e^{n-1},\xi^{n-1}\right){⫴}_{1}\leq \sigma {∥\left(e^{n-1},\xi^{n-1}\right)∥}_{1}\\ & \leq \sigma^{n}⫴\left(e^{0},\xi^{0}\right){⫴}_{1}\leq \sigma^{n}{∥F∥}_{-1}. \end{array}$$

From the discrete inf–sup condition (22), we have

(82) $$\begin{array}{cc} \beta_{0}{∥\eta^{n}∥}_{0} & \leq \frac{\lambda }{C_{\min}^{2}}\left(⫴\left(e^{n-1},\xi^{n-1}\right){⫴}_{1}⫴\left(u_{h},\omega_{h}\right){⫴}_{1}+⫴\left(e^{n},\xi^{n}\right){⫴}_{1}⫴\left(u_{h}^{n-1},\omega_{h}^{n-1}\right){⫴}_{1}\right)\\ & \;\;\;+\frac{C_{\max}}{C_{\min}}⫴\left(e^{n},\xi^{n}\right){⫴}_{1}\leq C\sigma^{n}{∥F∥}_{-1}. \end{array}$$

The proof is finished. □

In the next theorem, another expression of the error estimates will be given, which can be controlled by $\left(u_{h}^{n}-u_{h}^{n-1},\omega_{h}^{n}-\omega_{h}^{n-1}\right)$.

**Theorem** **8.** *Under the conditions of Theorems 5–7, there hold*

(83) $$\lim_{m\to +\infty }{∥\left(u_{h}^{m}-u_{h}^{m-1},\omega_{h}^{m}-\omega_{h}^{m-1}\right)∥}_{1}=0,$$

*and $\left(e^{m},\xi^{m}\right),\eta^{m}\left(m\geq 1\right))$ satisfy*

(84) $$⫴\left(e^{m},\xi^{m}\right){⫴}_{1}+∥\eta^{m}{∥}_{0}\leq C{∥\left(u_{h}^{m}-u_{h}^{m-1},\omega_{h}^{m}-\omega_{h}^{m-1}\right)∥}_{0}$$

*for the Stokes and Oseen iterative methods, and*

(85) $$⫴\left(e^{m},\xi^{m}\right){⫴}_{1}+∥\eta^{m}{∥}_{0}\leq c\left(h\right)∥\left(u_{h}^{m}-u_{h}^{m-1},\omega_{h}^{m}-\omega_{h}^{m-1}\right){∥}_{0}{∥\left(\nabla \left(u_{h}^{m}-u_{h}^{m-1}\right),\nabla \left(\omega_{h}^{m}-\omega_{h}^{m-1}\right)\right)∥}_{0}$$

*for the Newton iterative method.*

**Proof.**  With the help of the triangle inequality, we obtain

(86) $$∥\left(u_{h}^{m}-u_{h}^{m-1},\omega_{h}^{m}-\omega_{h}^{m-1}\right){∥}_{1}\leq ∥\left(e^{m},\xi^{m}\right){∥}_{1}+{∥\left(e^{m-1},\xi^{m-1}\right)∥}_{1}.$$

From Theorems 5–7, the Equation (83) can be derived.

Next, let us prove the Equation (84). Taking $\left(v,\psi \right)=\left(e^{m},\xi^{m}\right),q=\eta^{m}$ in (56) and using (12), we obtain

(87) $$\begin{array}{cc} & A\left(\left(e^{m},\xi^{m}\right),\left(e^{m},\xi^{m}\right)\right)+B\left(\left(e^{m},\xi^{m}\right),\left(u_{h},\omega_{h}\right),\left(e^{m},\xi^{m}\right)\right)\\ & \;\;\;\;=B(\left(u_{h}^{m-1},\xi_{h}^{m-1}\right),\left(e^{m-1}-e^{m},\xi^{m-1}-\xi^{m}\right),\left(e^{m},\xi^{m}\right))\\ & \;\;\;\;\;-B(\left(e^{m-1}-e^{m},\xi^{m-1}-\xi^{m}\right),\left(u_{h},\omega_{h}\right),\left(e^{m},\xi^{m}\right)). \end{array}$$

With the help of (11), (13), (29), (42) and (43), then

(88) $$\begin{array}{cc} & \frac{3}{5}⫴\left(e^{m},\xi^{m}\right){⫴}_{1}\leq \left(1-\sigma \right)⫴\left(e^{m},\xi^{m}\right){⫴}_{1}\\ & \;\;\;\;\leq C(∥\left(A_{h}u_{h},A_{h}\omega_{h}\right){∥}_{0}+∥\left(A_{h}u_{h}^{m-1},A_{h}\omega_{h}^{m-1}\right){∥}_{0})∥\left(e^{m-1}-e^{m},\xi^{m-1}-\xi^{m}\right){∥}_{0}\\ & \;\;\;\;\leq C∥\left(u_{h}^{m}-u_{h}^{m-1},\omega_{h}^{m}-\omega_{h}^{m-1}\right){∥}_{0}. \end{array}$$

Let $\left(v,\psi \right)=\left(e^{m},\xi^{m}\right),q=0$ in (56) and applying the discrete inf–sup condition (22), we arrive that

(89) $$\beta_{0}∥\eta^{m}{∥}_{0}\leq C⫴\left(e^{m},\xi^{m}\right){⫴}_{1}\leq C{∥\left(u_{h}^{m}-u_{h}^{m-1},\omega_{h}^{m}-\omega_{h}^{m-1}\right)∥}_{0}.$$

Thanks to (88) and (89), we obtain (84) for the Stokes iterative method.

Setting $\left(v,\psi \right)=\left(e^{m},\xi^{m}\right)$ and $q=\eta^{m}$ in (80) leads to

(90) $$\begin{array}{cc} & A\left(\left(e^{m},\xi^{m}\right),\left(e^{m},\xi^{m}\right)\right)+B\left(\left(e^{m},\xi^{m}\right),\left(u_{h},\omega_{h}\right),\left(e^{m},\xi^{m}\right)\right)\\ & \;\;\;\;=-B(\left(e^{m-1}-e^{m},\xi^{m-1}-\xi^{m}\right),\left(u_{h},\omega_{h}\right),\left(e^{m},\xi^{m}\right)). \end{array}$$

With the help of (11), (13), (29) and (76), we obtain

(91) $$\begin{array}{cc} \left(1-\sigma \right)⫴\left(e^{m},\xi^{m}\right){⫴}_{1} & \leq C∥\left(A_{h}u_{h},A_{h}\omega_{h}\right){∥}_{0}{∥\left(e^{m-1}-e^{m},\xi^{m-1}-\xi^{m}\right)∥}_{0}\\ & \leq C∥\left(u^{m}-u^{m-1},\omega^{m}-\omega^{m-1}\right){∥}_{0}. \end{array}$$

Setting $\left(v,\psi \right)=\left(e^{m},\xi^{m}\right),q=0$ in (80) and using the discrete inf-sup condition (22) yields

(92) $$\beta_{0}∥\eta^{m}{∥}_{0}\leq C{∥\left(u_{h}^{m}-u_{h}^{m-1},\omega_{h}^{m}-\omega_{h}^{m-1}\right)∥}_{0}.$$

Combining (88) and (89), the error estimate (84) for the Oseen iterative method is derived.

Finally, we prove the Equation (85). Choosing $\left(v,\psi \right)=\left(e^{m},\xi^{m}\right)$ and $q=\eta^{m}$ in (72) leads to

(93) $$\begin{array}{cc} & A\left(\left(e^{m},\xi^{m}\right),\left(e^{m},\xi^{m}\right)\right)+B\left(\left(e^{m},\xi^{m}\right),\left(u_{h},\omega_{h}\right),\left(e^{m},\xi^{m}\right)\right)\\ & \;\;\;\;=-B(\left(u_{h}^{m}-u_{h}^{m-1},\omega_{h}^{m}-\omega_{h}^{m-1}\right),\left(u_{h}^{m}-u_{h}^{m-1},\omega_{h}^{m}-\omega_{h}^{m-1}\right),\left(e^{m},\xi^{m}\right)). \end{array}$$

Using (11), (13), (29) and (59), we have

(94) $$\begin{array}{cc} & \frac{6}{11}⫴\left(e^{m},\xi^{m}\right){⫴}_{1}\leq \left(1-\sigma \right)⫴\left(e^{m},\xi^{m}\right){⫴}_{1}\\ & \;\;\;\;\leq c_{0}\left(h\right)∥\left(u_{h}^{m}-u_{h}^{m-1},\omega_{h}^{m}-\omega_{h}^{m-1}\right){∥}_{0}{∥\left(\nabla \left(u_{h}^{m}-u_{h}^{m-1}\right),\nabla \left(\xi_{h}^{m}-\xi_{h}^{m-1}\right)\right)∥}_{0}, \end{array}$$

where $c_{0}\left(h\right)=C{\left|\log h\right|}^{\frac{1}{2}}$ for d=2 and $c_{0}\left(h\right)=Ch^{-\frac{1}{2}}$ for d=3. Similarly, taking $\left(v,\psi \right)=\left(e^{m},\xi^{m}\right),q=0$ in (72) and using the discrete inf-sup condition (22), we have

(95) $$\begin{array}{cc} \beta_{0}{∥\eta^{m}∥}_{0} & \leq \frac{C_{\max}}{C_{\min}}⫴\left(e^{m},\xi^{m}\right){⫴}_{1}+\sigma ⫴\left(e^{m},\xi^{m}\right){⫴}_{1}\\ & \;\;\;+c_{0}\left(h\right)∥\left(u_{h}^{m}-u_{h}^{m-1},\xi_{h}^{m}-\xi_{h}^{m-1}\right){∥}_{0}{∥\left(\nabla \left(u_{h}^{m}-u_{h}^{m-1}\right),\nabla \left(\xi_{h}^{m}-\xi_{h}^{m-1}\right)\right)∥}_{0}\\ & \leq c\left(h\right)∥\left(u_{h}^{m}-u_{h}^{m-1},\omega_{h}^{m}-\omega_{h}^{m-1}\right){∥}_{0}{∥\left(\nabla \left(u_{h}^{m}-u_{h}^{m-1}\right),\nabla \left(\xi_{h}^{m}-\xi_{h}^{m-1}\right)\right)∥}_{0}. \end{array}$$

Thus, the (85) holds for the Newton iterative method and the proof ends. □

**Remark** **1.** *The error factor σ is not easy to compute during program implementation. Hence, we can adopt the error estimate from Theorem 8. In this case, the error can be controlled by the iterative error and the mesh size h.*

**Theorem** **9.** *Under the conditions of Theorems 3–7, the optimal error estimates of the three methods satisfy*

(96) $$⫴\left(u-u_{h}^{m},\omega -\omega_{h}^{m}\right){⫴}_{1}+{∥p-p_{h}^{m}∥}_{0}\leq C\left({h∥F∥}_{0}+{\left(\frac{11}{5}\sigma \right)}^{m}\frac{2}{5}{∥F∥}_{-1}\right),$$

*for the Stokes iterative method with*0<σ<2/5;

(97) $$⫴\left(u-u_{h}^{m},\omega -\omega_{h}^{m}\right){⫴}_{1}+{∥p-p_{h}^{m}∥}_{0}\leq C\left({h∥F∥}_{0}+{\left(\frac{15}{13}\sigma \right)}^{2^{m}-1}\frac{5}{11}{∥F∥}_{-1}\right),$$

*for the Newton iterative method with*0<σ<5/11;

(98) $$⫴\left(u-u_{h}^{m},\omega -\omega_{h}^{m}\right){⫴}_{1}+∥p-p_{h}^{m}{∥}_{0}\leq {C(h∥F∥}_{0}+\sigma^{m}{∥F∥}_{-1}),$$

*for the Oseen iterative method with 0<σ<1.*

## 5. Numerical Examples

In this section, four numerical examples are presented to test the correctness of the theoretical results and the validity of the proposed methods. In the first two examples, the three iterative methods are implemented for different viscosity coefficients. Meanwhile, the triangular cavity problem and bearing lubrication problem are also simulated in the last two examples. The P1b−P1−P1b finite element pair is used to approximate u,p and ω. We choose the public domain finite element software FreeFem++ [26] to implement the algorithms. For numerical implementations, the iterative tolerance is set as $1.0\times 10^{-6}$.

### 5.1. 2D/3D Problems with Exact Solutions

In this example, the 2D/3D problems with exact solutions are used to verify the rates of convergence. Set the fluid domain $\Omega ={\left[0,1\right]}^{d},d=2,3,\nu =\nu_{r}=c_{0}=c_{a}=c_{d}=1$ and choose the right-hand side functions *f* and *g* such that the analytical solutions are

$$\begin{array}{cc} u_{1} & =\pi \sin\left(2\pi y\right)\sin^{2}\left(\pi x\right),u_{2}=-\pi \sin\left(2\pi x\right)\sin^{2}\left(\pi y\right),\\ w & =\pi \sin^{2}\left(\pi x\right)\sin^{2}\left(\pi y\right),p=10\cos\left(\pi x\right)\cos\left(\pi y\right), \end{array}$$

for d=2 and

$$\begin{array}{cc} & u_{1}=-2\left(1-\cos\left(2\pi x\right)\right)\sin\left(2\pi y\right)\sin\left(2\pi z\right),\\ & u_{2}=\sin\left(2\pi x\right)\left(1-\cos\left(2\pi y\right)\right)\sin\left(2\pi z\right),\\ & u_{3}=\sin\left(2\pi x\right)\sin\left(2\pi y\right)\left(1-\cos\left(2\pi z\right)\right),\\ & w_{1}=\left(1-\cos\left(2\pi x\right)\right)\sin\left(2\pi y\right)\sin\left(2\pi z\right),\\ & w_{2}=\sin\left(2\pi x\right)\left(1-\cos\left(2\pi y\right)\right)\sin\left(2\pi z\right),\\ & w_{3}=\sin\left(2\pi x\right)\sin\left(2\pi y\right)\left(1-\cos\left(2\pi z\right)\right),\\ & p=10(\sin(4\pi x)+\sin(4\pi y)+\sin(4\pi z)), \end{array}$$

for d=3.

The CPU time and the convergence orders of the three iterative methods are displayed in Table 1, Table 2 and Table 3 for d=2, and Table A1, Table A2 and Table A3 for d=3. From these tables, we observe that the errors are almost the same with the three iterative methods. The corresponding errors are of the order of O(h), which accord with our theoretical analysis completely. In addition, from the data of the CPU time, the Newton iterative method is the most efficient when the mesh size is fixed. This is due to the Newton iterative method being of the convergence rate of the second order with respect to the iterative step *m*.

**Figure 1:**
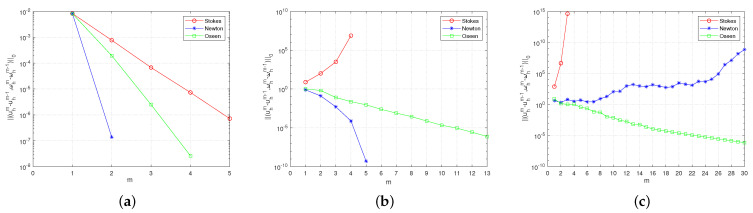
Comparison of iterative convergence errors with different viscosity coefficients. (**a**) $\nu =\nu_{r}=0.5\times 10^{0}$. (**b**) $\nu =\nu_{r}=0.5\times 10^{-2}$. (**c**) $\nu =\nu_{r}=0.5\times 10^{-3}$.

Figure 1 shows that in the 2D case, the convergence performance of the three iterative methods with different viscosity coefficients when the mesh size is fixed h=1/64. We can find that when the viscosity coefficients are small, the three methods are effective. As the viscosity coefficients increases, the Stokes iterative method is no longer applicable. When the viscosity coefficients $\nu =\nu_{r}=0.5\times 10^{-3}$, only the Oseen iteration can maintain convergence. The results are agreed with the ones predicted by the theoretical analysis. Since the results of 2D and 3D are similar, we present only the 2D results here. Please refer to the Appendix C for 3D results.

### 5.2. Driven Cavity Flow

In this example, the classical benchmark problem, known as driven cavity flow is considered. Let the domain $\Omega ={\left[0,1\right]}^{2},c_{a}=c_{d}=0.5,c_{0}=1,f=0,g=0$. The boundary condition for velocity reads u=(1,0) on y=1, and u=(0,0) on the other three boundaries. Meanwhile, the angular velocity satisfies the homogeneous Dirichlet boundary condition.

**Figure 2:**
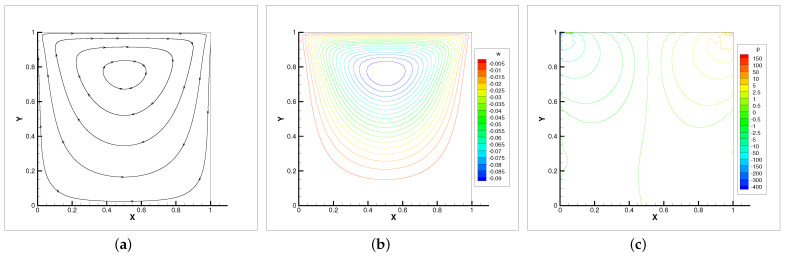
Velocity streamlines (**a**), angular velocity contour lines (**b**) and pressure isobars (**c**) with $\nu =\nu_{r}=0.5$.

**Figure 3:**
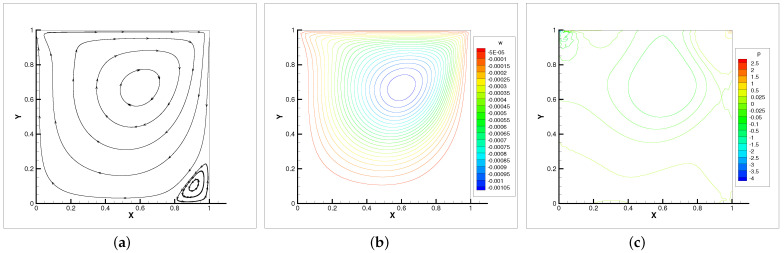
Velocity streamlines (**a**), angular velocity contour lines (**b**) and pressure isobars (**c**) with $\nu =\nu_{r}=0.005$.

**Figure 4:**
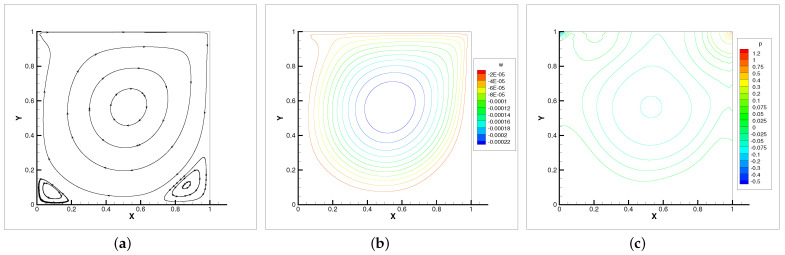
Velocity streamlines (**a**), angular velocity contour lines (**b**) and pressure isobars (**c**) with $\nu =\nu_{r}=0.001$.

In Table 4, we compare the applicability of the three iterative methods under different viscosity coefficients. As the table shows, the Oseen iteration is the most flexible method. This is consistent with our theoretical analysis. Thus, the Oseen iterative method is adopted. In Figure 2, Figure 3 and Figure 4, the velocity streamlines, angular velocity contour lines of $\omega =(0,0,\omega_{3})$ and pressure isobars for three different viscosity coefficients $\nu =\nu_{r}=0.5$, $\nu =\nu_{r}=0.005$, $\nu =\nu_{r}=0.001$ are displayed.

It can be seen that when the viscosity is small, the velocity streamlines, angular contour lines and pressure isobars satisfy symmetry. With the viscosity decreases, the main vortex center moves towards the geometric center of the cavity. Meanwhile, the secondary vortices appear near the corner of the cavity. The flow property of micropolar fluids is almost identical with the Navier–Stokes equations in [27].

### 5.3. Triangular Cavity Flow

**Figure A1:**
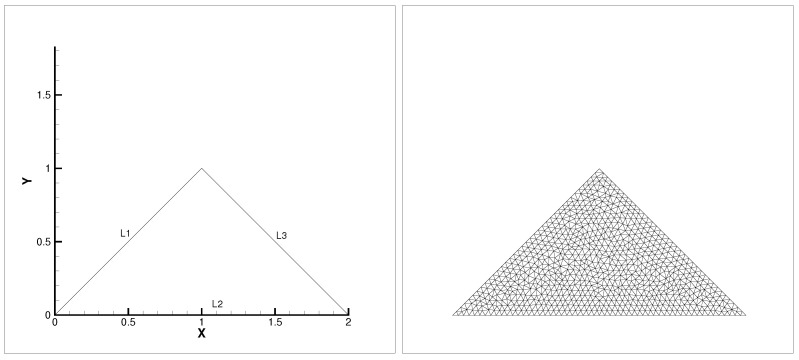
Study the geometry (**left**); Structured grid (**right**).

Motivated by [28], the flow of micropolar fluids inside an isosceles triangular cavity is considered. Figure A1 shows the schematic diagram of flow geometry and the computational mesh. Similarly, we only consider the Oseen iterative method in this example. Set $c_{a}=c_{d}=0.5,c_{0}=1,f=0,g=0$, the boundary conditions are as follows:

(99) $$\left\{\begin{array}{cc} u=(0,0), & \mathrm{on}L1\mathrm{and}L3,\\ u=(1,0), & \mathrm{on}L2,\\ \omega =0, & \mathrm{on}L1,L2,L3. \end{array}\right.$$

**Figure A2:**
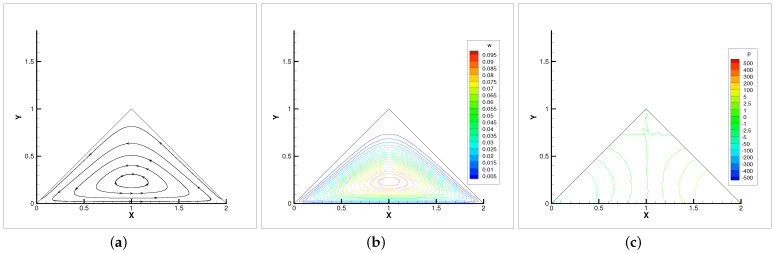
Velocity streamlines (**a**), angular velocity contour lines (**b**) and pressure isobars (**c**) with $\nu =\nu_{r}=0.5$.

**Figure A3:**
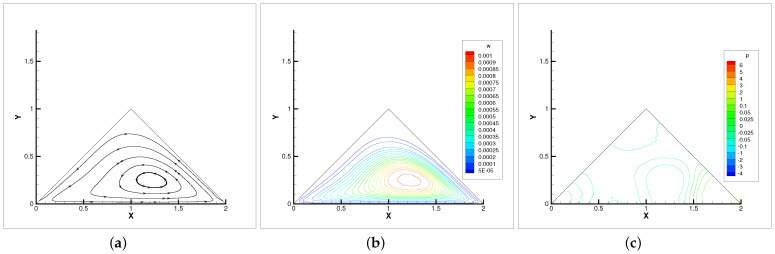
Velocity streamlines (**a**), angular velocity contour lines (**b**) and pressure isobars (**c**) with $\nu =\nu_{r}=0.005$.

**Figure A4:**
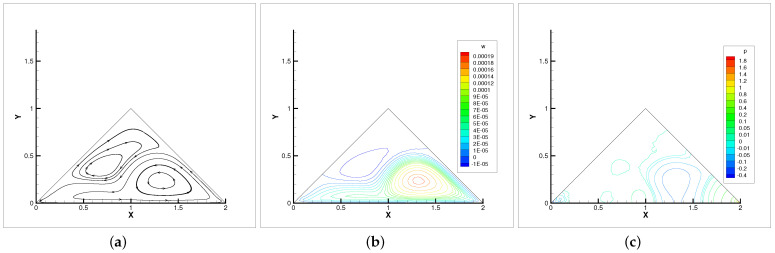
Velocity streamlines (**a**), angular velocity contour lines (**b**) and pressure isobars (**c**) with $\nu =\nu_{r}=0.001$.

Similar to square cavity flow, when the hydrodynamic viscosity decreases, the velocity streamlines, angular velocity contour lines and pressure isobars no longer satisfy symmetry. In summary, the flow property is still exhibited as laminar flow. Please refer to the Appendix D for Figure A2, Figure A3 and Figure A4.

### 5.4. The Bearing Lubrication Problem

In ordinary operating conditions, the lubricant liquid can be considered as fluid suspension. Thus the micropolar fluid equations can be used to simulate the lubrication problem [29]. In this example, the application of micropolar fluids in a non-concentric bearing lubrication problem is considered. The fluid region is a ring domain between the outer boundary $\Gamma_{1}$ with radius $r_{1}$ and the inner boundary $\Gamma_{2}$ with radius $r_{2}$.

We assume that velocity and angular velocity satisfy the homogeneous Dirichlet boundary condition at the boundary $\Gamma_{1}$. On the boundary $\Gamma_{2}$, the velocity and angular velocity satisfy $u_{1}=-\omega_{r}r_{2}\sin\theta$, $u_{2}=\omega_{r}r_{2}\cos\theta$ and ω=0. Here, $\omega_{r}$ represents the rotational angular velocity. Let $\nu =\nu_{r}=c_{a}=c_{d}=c_{0}=1$ and θ=arctan(y/x). We consider the three cases: $\omega_{r}$ = 200, 600, 1000.

**Figure 5:**
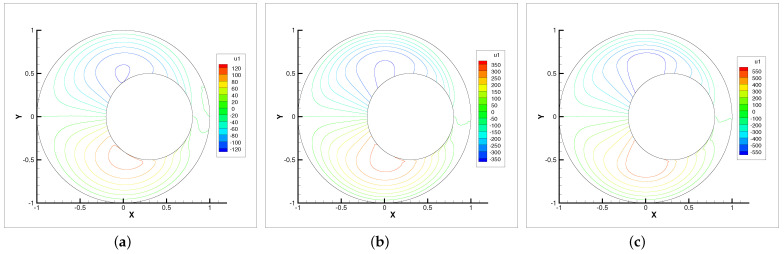
Horizontal velocity contour lines with $\omega_{r}=200,600,1000$. (**a**) $\omega_{r}=200$. (**b**) $\omega_{r}=600$. (**c**) $\omega_{r}=1000$.

**Figure 6:**
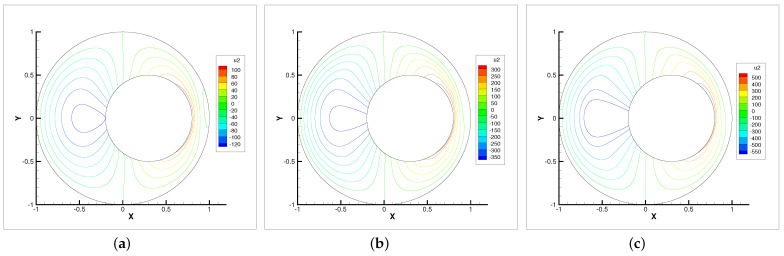
Vertical velocity contour lines with $\omega_{r}=200,600,1000$. (**a**) $\omega_{r}=200$. (**b**) $\omega_{r}=600$. (**c**) $\omega_{r}=1000$.

**Figure 7:**
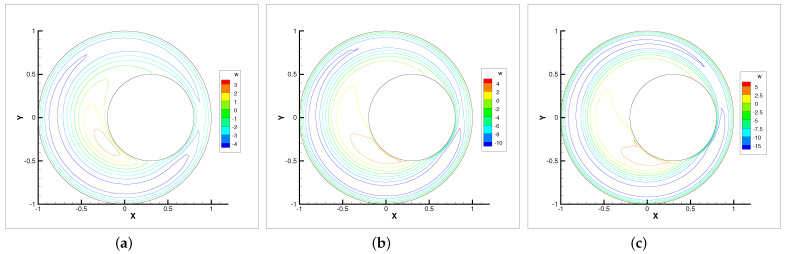
Angular velocity contour lines with $\omega_{r}=200,600,1000$. (**a**) $\omega_{r}=200$. (**b**) $\omega_{r}=600$. (**c**) $\omega_{r}=1000$.

**Figure 8:**
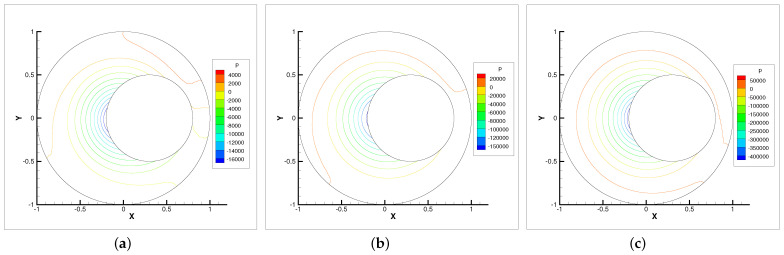
Pressure isobars with $\omega_{r}=200,600,1000$. (**a**) $\omega_{r}=200$. (**b**) $\omega_{r}=600$. (**c**) $\omega_{r}=1000$.

In this example, we only present the numerical experimental results for the Newton iterative method since it is faster than the Stokes and Oseen iterative methods. Figure 5, Figure 6, Figure 7 and Figure 8 show the evolution diagram of velocity components (horizontal velocity, vertical velocity), angular velocity and pressure at three different rotational angular velocities $\omega_{r}$.

The numerical results show that the velocity components, angular velocity and pressure increase with the increase in the rotational angular velocity $\omega_{r}$. The pressure increase is the most obvious. Therefore, the bearing is capable of supporting higher loads. The angular velocity contour lines tend to revolve around the inner circle. This means that the higher the rotational angular velocity $\omega_{r}$, the stronger the micropolarity effect of the fluid.

## 6. Conclusions

In this paper, three iterative methods are analyzed and compared for the micropolar fluid equations. The strong stability conditions for the Stokes and Newton iterations can be determined by the parameter σ, i.e., 0<σ<2/5 and 0<σ<5/11, respectively. Furthermore, the uniqueness condition 0<σ<1 for the Oseen iterative method is derived. Theoretical results and numerical simulations show that the iterative algorithm for the Stokes iteration is the simplest for larger viscosity coefficients, and the Newton iteration is the most efficient method for relatively large viscosity coefficients. The Oseen iterative method is the most flexible method because its stability and convergence condition is the weakest. Furthermore, the simplified bearing lubrication problem with inhomogeneous boundary conditions is considered and some interesting physical phenomena are observed. In addition, we notice that the results of pressure in the third example are not ideal. In future research, we will consider to improve pressure stability by adding stability terms.

## Figures and Tables

**Table 1:** Stokes iterative method for 2D stationary micropolar fluid equations.

$\frac{1}{h}$	CPU(s)	$\frac{∥\left(u-u_{h}\right){∥}_{1}}{{∥u∥}_{1}}$	Rate	$\frac{∥\left(u-u_{h}\right){∥}_{0}}{{∥u∥}_{0}}$	Rate	$\frac{∥\left(\omega -\omega_{h}\right){∥}_{1}}{{∥\omega ∥}_{1}}$	Rate	$\frac{∥\left(\omega -\omega_{h}\right){∥}_{0}}{{∥\omega ∥}_{0}}$	Rate	$\frac{∥p-p_{h}{∥}_{0}}{{∥p∥}_{0}}$	Rate
8	0.186	$3.01\times 10^{-1}$	0	$1.14\times 10^{-1}$	0	$2.67\times 10^{-1}$	0	$8.35\times 10^{-2}$	0	$4.06\times 10^{-1}$	0
16	0.605	$1.52\times 10^{-1}$	0.99	$2.95\times 10^{-2}$	1.95	$1.36\times 10^{-1}$	0.97	$2.22\times 10^{-2}$	1.91	$1.28\times 10^{-1}$	1.66
32	2.672	$7.58\times 10^{-2}$	1.00	$7.42\times 10^{-3}$	1.99	$6.85\times 10^{-2}$	0.99	$5.63\times 10^{-3}$	1.98	$4.24\times 10^{-2}$	1.60
64	11.058	$3.78\times 10^{-2}$	1.00	$1.85\times 10^{-3}$	2.00	$3.43\times 10^{-2}$	1.00	$1.41\times 10^{-3}$	1.99	$1.46\times 10^{-2}$	1.54
128	51.860	$1.89\times 10^{-2}$	1.00	$4.62\times 10^{-4}$	2.00	$1.72\times 10^{-2}$	1.00	$3.53\times 10^{-4}$	2.00	$5.09\times 10^{-3}$	1.52

**Table 2:** Newton iterative method for 2D stationary micropolar fluid equations.

$\frac{1}{h}$	CPU(s)	$\frac{∥\left(u-u_{h}\right){∥}_{1}}{{∥u∥}_{1}}$	Rate	$\frac{∥\left(u-u_{h}\right){∥}_{0}}{{∥u∥}_{0}}$	Rate	$\frac{∥\left(\omega -\omega_{h}\right){∥}_{1}}{{∥\omega ∥}_{1}}$	Rate	$\frac{∥\left(\omega -\omega_{h}\right){∥}_{0}}{{∥\omega ∥}_{0}}$	Rate	$\frac{∥p-p_{h}{∥}_{0}}{{∥p∥}_{0}}$	Rate
8	0.107	$3.01\times 10^{-1}$	0	$1.14\times 10^{-1}$	0	$2.67\times 10^{-1}$	0	$8.35\times 10^{-2}$	0	$4.06\times 10^{-1}$	0
16	0.351	$1.52\times 10^{-1}$	0.99	$2.95\times 10^{-2}$	1.95	$1.36\times 10^{-1}$	0.97	$2.22\times 10^{-2}$	1.91	$1.28\times 10^{-1}$	1.66
32	1.610	$7.58\times 10^{-2}$	1.00	$7.42\times 10^{-3}$	1.99	$6.85\times 10^{-2}$	0.99	$5.63\times 10^{-3}$	1.98	$4.24\times 10^{-2}$	1.60
64	6.635	$3.78\times 10^{-2}$	1.00	$1.85\times 10^{-3}$	2.00	$3.43\times 10^{-2}$	1.00	$1.41\times 10^{-3}$	1.99	$1.46\times 10^{-2}$	1.54
128	29.636	$1.89\times 10^{-2}$	1.00	$4.62\times 10^{-4}$	2.00	$1.72\times 10^{-2}$	1.00	$3.53\times 10^{-4}$	2.00	$5.09\times 10^{-3}$	1.52

**Table 3:** Oseen iterative method for 2D stationary micropolar fluid equations.

$\frac{1}{h}$	CPU(s)	$\frac{∥\left(u-u_{h}\right){∥}_{1}}{{∥u∥}_{1}}$	Rate	$\frac{∥\left(u-u_{h}\right){∥}_{0}}{{∥u∥}_{0}}$	Rate	$\frac{∥\left(\omega -\omega_{h}\right){∥}_{1}}{{∥\omega ∥}_{1}}$	Rate	$\frac{∥\left(\omega -\omega_{h}\right){∥}_{0}}{{∥\omega ∥}_{0}}$	Rate	$\frac{∥p-p_{h}{∥}_{0}}{{∥p∥}_{0}}$	Rate
8	0.167	$3.01\times 10^{-1}$	0	$1.14\times 10^{-1}$	0	$2.67\times 10^{-1}$	0	$8.35\times 10^{-2}$	0	$4.06\times 10^{-1}$	0
16	0.536	$1.52\times 10^{-1}$	0.99	$2.95\times 10^{-2}$	1.95	$1.36\times 10^{-1}$	0.97	$2.22\times 10^{-2}$	1.91	$1.28\times 10^{-1}$	1.66
32	2.349	$7.58\times 10^{-2}$	1.00	$7.42\times 10^{-3}$	1.99	$6.85\times 10^{-2}$	0.99	$5.63\times 10^{-3}$	1.98	$4.24\times 10^{-2}$	1.60
64	9.831	$3.78\times 10^{-2}$	1.00	$1.85\times 10^{-3}$	2.00	$3.43\times 10^{-2}$	1.00	$1.41\times 10^{-3}$	1.99	$1.46\times 10^{-2}$	1.54
128	44.989	$1.89\times 10^{-2}$	1.00	$4.62\times 10^{-4}$	2.00	$1.72\times 10^{-2}$	1.00	$3.53\times 10^{-4}$	2.00	$5.09\times 10^{-3}$	1.52

**Table 4:** Comparison of the iterative numbers.

Iterative Method	$\nu =\nu_{r}=0.5\times 10^{0}$	$\nu =\nu_{r}=0.5\times 10^{-2}$	$\nu =\nu_{r}=0.1\times 10^{-2}$
Stokes	5	—	—
Newton	3	6	—
Oseen	3	13	22

## Data Availability

Not applicable.

## Appendix A

**The** **Proof** **of** **Theorem** **2.** The weak form of (9) can be written as

(A1) $$\begin{array}{cc} & a\left(u,v\right)-d\left(\left(v,\psi \right),p\right)+d\left(\left(u,\omega \right),q\right)=\bar{c}\left(\omega ,v\right)+\langle f,v\rangle -b\left(u,u,v\right), \end{array}$$

(A2) $$\begin{array}{cc} & \bar{a}\left(\omega ,\psi \right)-\bar{k}\left(\omega ,\psi \right)=c\left(u,\psi \right)+\langle g,\psi \rangle -\bar{r}\left(\omega ,\psi \right)-\bar{b}(u,\omega ,\psi ). \end{array}$$

For Equation (A1), using the $H_{2}$ regularity of the Stokes problem [18] and Young’s inequality, we have

(A3) $$\begin{array}{cc} \nu_{0}{∥u∥}_{2}+{∥p∥}_{1} & \leq C(∥f{∥}_{0}+∥u{∥}_{L^{\infty }}∥\nabla u{∥}_{0}+2\nu_{r}∥\nabla \times \omega {∥}_{0})\\ & \leq C(∥f{∥}_{0}+∥u{∥}_{2}^{\frac{1}{2}}∥\nabla u{∥}_{0}^{\frac{3}{2}}+2\nu_{r}∥\nabla \omega {∥}_{0})\\ & \leq \frac{\nu_{0}}{2}{∥u∥}_{2}+{C(∥f∥}_{0}+{∥u∥}_{1}^{3}+2\nu_{r}{∥\omega ∥}_{1}). \end{array}$$

For Equation (A2), applying the $H_{2}$ regularity of the Poisson equation and Young’s inequality, we obtain

(A4) $$\begin{array}{cc} \left(c_{1}-c_{2}d\right){∥\omega ∥}_{2} & \leq c_{1}{∥\omega ∥}_{2}-c_{2}{∥\nabla \nabla \cdot \omega ∥}_{0}\\ & \leq C(∥g{∥}_{0}+2\nu_{r}∥\nabla \times u{∥}_{0}+∥\nabla u{∥}_{0}∥\omega {∥}_{L^{\infty }}+4\nu_{r}∥\omega {∥}_{0})\\ & \leq C(∥g{∥}_{0}+2\nu_{r}∥u{∥}_{1}+∥\nabla u{∥}_{0}∥\omega {∥}_{1}^{\frac{1}{2}}∥\omega {∥}_{2}^{\frac{1}{2}}+4\nu_{r}∥\omega {∥}_{1})\\ & \leq \frac{c_{1}-c_{2}d}{2}{∥\omega ∥}_{2}+{C(∥g∥}_{0}+2\nu_{r}{∥u∥}_{1}+{∥u∥}_{1}^{2}{∥\omega ∥}_{1}+4\nu_{r}{∥\omega ∥}_{1}). \end{array}$$

Finally, combining (A3) with (A4). The proof is completed. □

## Appendix B

**The** **Proof** **of** **Theorem** **4.** Subtracting (26) from (9), we get the following error equation

(A5) $$\begin{array}{cc} & A\left(\left(u-u_{h},\omega -\omega_{h}\right),\left(v,\psi \right)\right)+B\left(\left(u-u_{h},\omega -\omega_{h}\right),\left(u,\omega \right),\left(v,\psi \right)\right)\\ & \;\;\;\;+B(\left(u_{h},\omega_{h}\right),\left(u-u_{h},\omega -\omega_{h}\right),\left(v,\psi \right))\\ & \;\;\;\;+d\left(\left(u-u_{h},\omega -\omega_{h}\right),q\right)-d\left(\left(v,\psi \right),p-p_{h}\right)=0. \end{array}$$

Let $\left(e,\xi \right)=\left(R_{h}u-u_{h},Q_{h}\omega -\omega_{h}\right)$ and $\eta =\rho_{h}p-p_{h}$, choosing (v,ψ)=(e,ξ),q=η in (26) and combining (12), we have

(A6) $$\begin{array}{cc} & A((e,\xi ),(e,\xi ))+B((e,\xi ),(u,\omega ),(e,\xi ))\\ & \;\;\;\;=A\left(\left(R_{h}u-u,Q_{h}\omega -\omega \right),\left(e,\xi \right)\right)+B\left(\left(R_{h}u-u,Q_{h}\omega -\omega \right),\left(u,\omega \right),\left(e,\xi \right)\right)\\ & \;\;\;\;\;\;\;\;+B(\left(u_{h},\omega_{h}\right),\left(R_{h}u-u,Q_{h}\omega -\omega \right),\left(e,\xi \right))\\ & \;\;\;\;\;\;\;\;+d\left(\left(e,\xi \right),p-p_{h}\right)-d\left(\left(u-u_{h},\omega -\omega_{h}\right),\rho_{h}p-p_{h}\right). \end{array}$$

On the other hand,

(A7) $$d\left(\left(e,\xi \right),p-p_{h}\right)-d\left(\left(u-u_{h},\omega -\omega_{h}\right),\rho_{h}p-p_{h}\right)=d\left(\left(e,\xi \right),p-\rho_{h}p\right).$$

Applying (11), (13) and (15), the left-hand side of (A6) can be bounded by

(A8) $$l.h.s\geq C_{\min}{∥\left(e,\xi \right)∥}_{1}^{2}-{\lambda ∥\left(u,\omega \right)∥}_{1}{∥\left(e,\xi \right)∥}_{1}^{2}\geq C_{\min}\left(1-\sigma \right){∥\left(e,\xi \right)∥}_{1}^{2}.$$

Similarly, the right-hand side of (A6) can be bounded by

(A9) $$\begin{array}{cc} r.h.s & \leq {∥\left(e,\xi \right)∥}_{1}[∥\left(R_{h}u-u,Q_{h}\omega -\omega \right){∥}_{1}(C_{\max}+\lambda ∥\left(u,\omega \right){∥}_{1}+\lambda ∥\left(u_{h},\omega_{h}\right){∥}_{1})\\ & \;\;\;\;+∥p-\rho_{h}p{∥}_{0}]\\ & \leq {∥\left(e,\xi \right)∥}_{1}[∥\left(R_{h}u-u,Q_{h}\omega -\omega \right){∥}_{1}\left(C_{\max}+2C_{\min}\sigma \right)+∥p-\rho_{h}p{∥}_{0}]\\ & \leq {C∥\left(e,\xi \right)∥}_{1}(∥\left(R_{h}u-u,Q_{h}\omega -\omega \right){∥}_{1}+∥p-\rho_{h}p{∥}_{0}). \end{array}$$

Combining the above two inequalities yields

(A10) $$⫴\left(u-u_{h},\omega -\omega_{h}\right){⫴}_{1}\leq C(∥\left(R_{h}u-u,Q_{h}\omega -\omega \right){∥}_{1}+∥p-\rho_{h}p{∥}_{0}).$$

Let q=0 in (A5) leads to

(A11) $$\begin{array}{cc} d(\left(v,\psi \right),p-p_{h}) & =A\left(\left(u-u_{h},\omega -\omega_{h}\right),\left(v,\psi \right)\right)+B\left(\left(u-u_{h},\omega -\omega_{h}\right),\left(u,\omega \right),\left(v,\psi \right)\right)\\ & \;\;\;+B(\left(u_{h},\omega_{h}\right),\left(u-u_{h},\omega -\omega_{h}\right),\left(v,\psi \right)). \end{array}$$

With the help of discrete inf-sup condition (22), we obtain

(A12) $$\begin{array}{cc} \beta_{0}{∥p-p_{h}∥}_{0} & \leq (C_{\max}+{\lambda ∥\left(u,\omega \right)∥}_{1}+\lambda ∥\left(u_{h},\omega_{h}\right){∥}_{1})∥\left(u-u_{h},\omega -\omega_{h}\right){∥}_{1}\\ & \leq \left(\frac{C_{\max}}{C_{\min}}+2\sigma \right)⫴\left(u-u_{h},\omega -\omega_{h}\right){⫴}_{1}\\ & \leq C⫴\left(u-u_{h},\omega -\omega_{h}\right){⫴}_{1}. \end{array}$$

From (A10) and (A12), we conclude that

(A13) $$⫴\left(u-u_{h},\omega -\omega_{h}\right){⫴}_{1}+{∥p-p_{h}∥}_{0}\leq Ch.$$

Then, let us prove $L^{2}{\left(\Omega \right)}^{d}$ norm error estimate. Set the dual problem [6,18] as follows: seek ((w,ϕ),s)∈W×M such that

(A14) $$\begin{array}{cc} & A((v,\psi ),(w,\phi ))+B((u,\omega ),(v,\psi ),(w,\phi ))+B((v,\psi ),(u,\omega ),(w,\phi ))-d((v,\psi ),s)\\ & \;\;\;\;+d\left(\left(w,\phi \right),q\right)=\left(\left(u-u_{h},\omega -\omega_{h}\right),\left(v,\psi \right)\right),\forall \left(\left(v,\psi \right),q\right)\in W\times M. \end{array}$$

If the solution of (A14) satisfies $w,\phi \in H^{2}{\left(\Omega \right)}^{d}\cap X$, and $s\in H^{1}\left(\Omega \right)\cap M$, this means that

(A15) $${∥\left(w,\phi \right)∥}_{2}+{∥s∥}_{1}\leq C{∥\left(u-u_{h},\omega -\omega_{h}\right)∥}_{0}.$$

Let $\left(v,\psi \right)=\left(u-u_{h},\omega -\omega_{h}\right),q=0$ in (A14) and subtract it from (26) with q=0, we get

(A16) $$\begin{array}{cc} & ∥\left(u-u_{h},\omega -\omega_{h}\right){∥}_{0}^{2}=A\left(\left(u-u_{h},\omega -\omega_{h}\right),\left(w-v,\phi -\psi \right)\right)\\ & \;\;\;\;+B(\left(u-u_{h},\omega -\omega_{h}\right),\left(u,\omega \right),\left(w-v,\phi -\psi \right))\\ & \;\;\;\;+B(\left(u_{h},\omega_{h}\right),\left(u-u_{h},\omega -\omega_{h}\right),\left(w-v,\phi -\psi \right))\\ & \;\;\;\;-d\left(\left(w-v,\phi -\psi \right),p-p_{h}\right)-d\left(\left(u-u_{h},\omega -\omega_{h}\right),s-\rho_{h}s\right). \end{array}$$

Setting $v_{h}=R_{h}w,\psi_{h}=Q_{h}\phi$ and using (10), (13), (16) and (A15), we arrive that

(A17) $$\begin{array}{cc} & ∥\left(u-u_{h},\omega -\omega_{h}\right){∥}_{0}^{2}\\ & \;\;\;\;\leq C_{\max}∥\left(u-u_{h},\omega -\omega_{h}\right){∥}_{1}{∥\left(w-R_{h}w,\phi -Q_{h}\phi \right)∥}_{1}\\ & \;\;\;\;\;\;\;\;+{\lambda (∥\left(u,\omega \right)∥}_{1}+∥\left(u_{h},\omega_{h}\right){∥}_{1})∥\left(u-u_{h},\omega -\omega_{h}\right){∥}_{1}{∥\left(w-R_{h}w,\phi -Q_{h}\phi \right)∥}_{1}\\ & \;\;\;\;\;\;\;\;+∥\left(u-u_{h},\omega -\omega_{h}\right){∥}_{1}∥s-\rho_{h}{s∥}_{0}+∥\left(w-R_{h}w,\phi -Q_{h}\phi \right){∥}_{1}{∥p-p_{h}∥}_{0}\\ & \;\;\;\;\leq Ch\left(1+\sigma \right)⫴\left(u-u_{h},\omega -\omega_{h}\right){⫴}_{1}⫴\left(w,\phi \right){⫴}_{2}\\ & \;\;\;\;\;\;\;\;+Ch(⫴\left(u-u_{h},\omega -\omega_{h}\right){⫴}_{1}∥s{∥}_{1}+⫴\left(w,\phi \right){⫴}_{2}∥p-p_{h}{∥}_{0})\\ & \;\;\;\;\leq Ch(⫴\left(u-u_{h},\omega -\omega_{h}\right){⫴}_{1}+∥p-p_{h}{∥}_{0})∥\left(u-u_{h},\omega -\omega_{h}\right){∥}_{0}, \end{array}$$

which indicates that

(A18) $$∥\left(u-u_{h},\omega -\omega_{h}\right){∥}_{0}\leq Ch(⫴\left(u-u_{h},\omega -\omega_{h}\right){⫴}_{1}+∥p-p_{h}{∥}_{0}).$$

The proof is completed. □

## Appendix C. The Tables of 2D/3D Problems with Exact Solutions

**Table A1:** Stokes iterative method for 3D stationary micropolar fluid equations.

$\frac{1}{h}$	CPU(s)	$\frac{∥\left(u-u_{h}\right){∥}_{1}}{{∥u∥}_{1}}$	Rate	$\frac{∥\left(u-u_{h}\right){∥}_{0}}{{∥u∥}_{0}}$	Rate	$\frac{∥\left(\omega -\omega_{h}\right){∥}_{1}}{{∥\omega ∥}_{1}}$	Rate	$\frac{∥\left(\omega -\omega_{h}\right){∥}_{0}}{{∥\omega ∥}_{0}}$	Rate	$\frac{∥p-p_{h}{∥}_{0}}{{∥p∥}_{0}}$	Rate
4	2.177	$7.51\times 10^{-1}$	0	$5.93\times 10^{-1}$	0	$7.13\times 10^{-1}$	0	$5.29\times 10^{-1}$	0	$2.03\times 10^{0}$	0
8	21.445	$4.24\times 10^{-1}$	0.82	$2.06\times 10^{-1}$	1.52	$4.16\times 10^{-1}$	0.78	$1.78\times 10^{-1}$	1.57	$7.01\times 10^{-1}$	1.53
12	78.571	$2.89\times 10^{-1}$	0.95	$9.72\times 10^{-2}$	1.85	$2.86\times 10^{-1}$	0.92	$8.41\times 10^{-2}$	1.86	$3.62\times 10^{-1}$	1.63
16	207.854	$2.18\times 10^{-1}$	0.97	$5.58\times 10^{-2}$	1.93	$2.17\times 10^{-1}$	0.96	$4.83\times 10^{-2}$	1.93	$2.29\times 10^{-1}$	1.59
20	421.663	$1.75\times 10^{-1}$	0.99	$3.61\times 10^{-2}$	1.96	$1.74\times 10^{-1}$	0.98	$3.12\times 10^{-2}$	1.96	$1.60\times 10^{-1}$	1.59

**Table A2:** Newton iterative method for 3D stationary micropolar fluid equations.

$\frac{1}{h}$	CPU(s)	$\frac{∥\left(u-u_{h}\right){∥}_{1}}{{∥u∥}_{1}}$	Rate	$\frac{∥\left(u-u_{h}\right){∥}_{0}}{{∥u∥}_{0}}$	Rate	$\frac{∥\left(\omega -\omega_{h}\right){∥}_{1}}{{∥\omega ∥}_{1}}$	Rate	$\frac{∥\left(\omega -\omega_{h}\right){∥}_{0}}{{∥\omega ∥}_{0}}$	Rate	$\frac{∥p-p_{h}{∥}_{0}}{{∥p∥}_{0}}$	Rate
4	1.523	$7.51\times 10^{-1}$	0	$5.93\times 10^{-1}$	0	$7.13\times 10^{-1}$	0	$5.29\times 10^{-1}$	0	$2.03\times 10^{0}$	0
8	16.878	$4.24\times 10^{-1}$	0.82	$2.06\times 10^{-1}$	1.52	$4.16\times 10^{-1}$	0.78	$1.78\times 10^{-1}$	1.57	$7.01\times 10^{-1}$	1.53
12	61.688	$2.89\times 10^{-1}$	0.95	$9.72\times 10^{-2}$	1.85	$2.86\times 10^{-1}$	0.92	$8.41\times 10^{-2}$	1.86	$3.62\times 10^{-1}$	1.63
16	161.407	$2.18\times 10^{-1}$	0.97	$5.58\times 10^{-2}$	1.93	$2.17\times 10^{-1}$	0.96	$4.83\times 10^{-2}$	1.93	$2.29\times 10^{-1}$	1.59
20	327.385	$1.75\times 10^{-1}$	0.99	$3.61\times 10^{-2}$	1.96	$1.74\times 10^{-1}$	0.98	$3.12\times 10^{-2}$	1.96	$1.60\times 10^{-1}$	1.59

**Table A3:** Oseen iterative method for 3D stationary micropolar fluid equations.

$\frac{1}{h}$	CPU(s)	$\frac{∥\left(u-u_{h}\right){∥}_{1}}{{∥u∥}_{1}}$	Rate	$\frac{∥\left(u-u_{h}\right){∥}_{0}}{{∥u∥}_{0}}$	Rate	$\frac{∥\left(\omega -\omega_{h}\right){∥}_{1}}{{∥\omega ∥}_{1}}$	Rate	$\frac{∥\left(\omega -\omega_{h}\right){∥}_{0}}{{∥\omega ∥}_{0}}$	Rate	$\frac{∥p-p_{h}{∥}_{0}}{{∥p∥}_{0}}$	Rate
4	1.865	$7.51\times 10^{-1}$	0	$5.93\times 10^{-1}$	0	$7.13\times 10^{-1}$	0	$5.29\times 10^{-1}$	0	$2.03\times 10^{0}$	0
8	19.234	$4.24\times 10^{-1}$	0.82	$2.06\times 10^{-1}$	1.52	$4.16\times 10^{-1}$	0.78	$1.78\times 10^{-1}$	1.57	$7.01\times 10^{-1}$	1.53
12	89.336	$2.89\times 10^{-1}$	0.95	$9.72\times 10^{-2}$	1.85	$2.86\times 10^{-1}$	0.92	$8.41\times 10^{-2}$	1.86	$3.62\times 10^{-1}$	1.63
16	231.013	$2.18\times 10^{-1}$	0.97	$5.58\times 10^{-2}$	1.93	$2.17\times 10^{-1}$	0.96	$4.83\times 10^{-2}$	1.93	$2.29\times 10^{-1}$	1.59
20	417.197	$1.75\times 10^{-1}$	0.99	$3.61\times 10^{-2}$	1.96	$1.74\times 10^{-1}$	0.98	$3.12\times 10^{-2}$	1.96	$1.60\times 10^{-1}$	1.59
